# Mechanism-Guided Design of a PPy–TiO_2_–CNT Nanocomposite for Visible-Light Photodegradation

**DOI:** 10.1021/acsomega.6c02369

**Published:** 2026-06-10

**Authors:** Al-Ali Hussein, Soon Huat Tan, Vel Murugan Vadivelu

**Affiliations:** School of Chemical Engineering, Universiti Sains Malaysia, Engineering Campus, 14300 Nibong Tebal, Pulau Pinang, Malaysia

## Abstract

Visible-light dye photodegradation is often constrained by band-structure limitations, weak interfacial coupling, and slow charge transport, which collectively accelerate electron–hole recombination and limit durability. To address these bottlenecks, a polypyrrole–titanium dioxide–carbon nanotube (PPy–TiO_2_–CNT) heterojunction was designed in which PPy serves as a visible-light photosensitizer, TiO_2_ provides a stable oxidative scaffold and electron-accepting phase, and CNTs create conductive pathways that strengthen interfacial coupling and accelerate electron extraction and transfer. Unlike previously reported binary PPy–oxide or PPy/CNT systems, the present work develops a continuously coupled ternary PPy–TiO_2_–CNT interface that integrates visible-light sensitization, oxide redox functionality, and CNT-mediated electron conduction within a single heterojunction while also linking the material design to an LC–MS-verified degradation pathway. The synthesized PPy–TiO_2_–CNT heterojunction nanocomposite was evaluated by systematic screening of key operating variables (pH 3–11; 5–50 mg L^–1^; 0.10–1.00 mg mL^–1^). Under optimal conditions, the nanocomposite achieved a rate constant of 0.053 min^–1^ and 99.4% MB removal under visible light, while retaining 92.3% activity after five cycles. The targeted bottlenecks were addressed experimentally by (i) extended visible absorption and an apparent optical gap of ≈2.10 eV (DRS/Tauc) to enhance photon harvesting, (ii) strong PL quenching, which provides evidence of suppressed radiative recombination, and (iii) the smallest Nyquist semicircle (lowest Rct) to confirm accelerated interfacial charge transfer. These characterization results collectively indicate improved interfacial charge separation and transport, which is reflected in the favorable quantitative performance of the optimized CTP-3 sample, namely an apparent optical gap of ≈2.10 eV, a rate constant of 0.053 min^–1^, 99.4% MB removal, and 92.3% retained activity after five cycles. Under visible irradiation, PPy acts as the primary photoactive component (HOMO → LUMO), while the CNT network promotes electron extraction and delivery to interfacial O_2_, supporting a reactive oxygen species (ROS) sequence from superoxide (^•^O_2_^–^) to hydroxyl radical (^•^OH) that oxidizes MB. Liquid chromatography–mass spectrometry reveals sequential N-demethylation, aromatic oxidation, and ring opening toward mineralization. Overall, the continuously coupled PPy–TiO_2_–CNT interface mitigates recombination and transport limitations while maintaining durability, establishing a reproducible route to mechanism-guided materials-process codesign for efficient visible-light photocatalysis.

## Graphical abstract

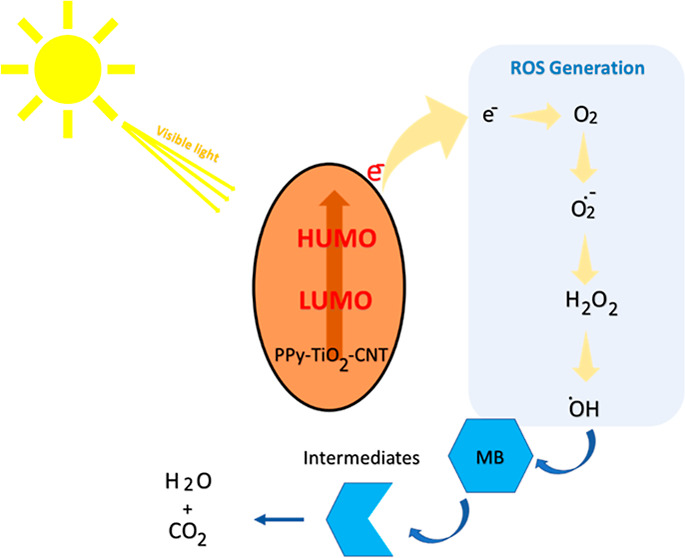

## 1 Introduction

Organic dye pollution, particularly methylene blue (MB), poses a severe environmental threat due to its extensive use in the textile, pharmaceutical, cosmetic, and paper industries. Industrial dyeing processes often result in significant MB contamination in aquatic systems, with an estimated 10–15% of dye loss directly entering waterways.^1,2^ Its persistence in aquatic ecosystems disrupts delicate ecological balances by inhibiting photosynthesis in aquatic plants and elevating chemical oxygen demand (COD) and biological oxygen demand (BOD), leading to oxygen depletion.^1,2^ Furthermore, exposure to MB poses significant human health risks, including reported toxic effects and irritation, depending on dose and exposure route.^2,3^ Conventional treatment methods such as adsorption, chemical oxidation, and biological degradation often fail to achieve complete pollutant removal, can introduce secondary pollution, and incur substantial operational costs.^4,5^

Adsorption, for instance, generates secondary waste and loses efficiency as adsorbents become saturated,^5,6^ while eco-friendly biological methods require long treatment times and are less effective against chemically stable pollutants like MB.^7^

Consequently, MB has been widely adopted as a model pollutant in studies aimed at the development of more effective treatment technologies. Among these, advanced oxidation processes (AOPs), especially heterogeneous photocatalysis, have emerged as promising alternatives capable of mineralizing MB into harmless byproducts such as CO_2_ and H_2_O.^1,7,8^ While semiconductor photocatalysts, such as titanium dioxide (TiO_2_), show strong oxidative capabilities, their practical application is restricted by ultraviolet (UV) activation, rapid electron–hole recombination, and low quantum efficiency.^2,3^

To address these limitations, the development of highly efficient and sustainable photocatalytic systems is paramount. At the forefront of this endeavor is the exploration of conductive polymers, with polypyrrole (PPy) emerging as a particularly compelling candidate. Conductive polymers are introduced to overcome two key bottlenecks associated with conventional semiconductors such as TiO_2_: limited visible-light absorption and rapid charge-carrier recombination.^9,10^ PPy’s inherent π-conjugated structure endows it with strong visible-light absorption and remarkable photosensitization capabilities, making it an ideal platform for harnessing solar energy.^11,12^ In addition, its electrical conductivity enables PPy to act as an interfacial charge-transport medium, promoting electron migration and suppressing recombination, which improves the utilization of photogenerated carriers in redox reactions.^10,13^ Moreover, PPy can be synthesized via simple oxidative polymerization under mild conditions, enabling scalable integration with oxide and carbon scaffolds. However, to unlock its full potential, a strategic hybridization with other functional nanomaterials is essential. This has led to a focus on developing advanced nanocomposites where PPy is synergistically integrated with materials such as TiO_2_ and carbon nanotubes (CNTs) to create a new generation of high-efficiency, visible-light-driven photocatalysts.

The rationale for constructing these PPy-centric nanocomposites is based on a role-based selection of constituents and their engineered integration into a heterojunction architecture, in which each component addresses a specific limitation of the others. PPy serves as the primary visible-light photosensitizer; however, its photocatalytic performance is markedly improved when coupled with suitable semiconductors to promote charge separation and suppress recombination.^14^ TiO_2_ is incorporated for its chemical stability and oxidative functionality, acting as an electron-accepting scaffold that supports interfacial charge separation.^15,16^ To further address charge transport limitations, CNTs are introduced. As efficient electron mediators, CNTs create conductive pathways that accelerate the migration of photogenerated electrons and suppress their recombination with holes, which is critical for boosting photocatalytic efficiency and stability.^17–19^

In photocatalytic systems, these interfacial and conductive functions are especially important because charge carriers generated inside the light-harvesting phase must be extracted and transferred to reactive surface sites before recombination occurs. Thus, photocatalytic efficiency is governed not only by visible-light absorption but also by the extent of interfacial coupling, the directionality of charge transfer, and the continuity of conductive pathways across the heterojunction. This interpretation is supported by prior PPy–TiO_2_ studies. For example, in Polypyrrole/TiO_2_, PPy–TiO_2_ composites were shown to possess a narrowed band gap, improved visible-light absorption, and favorable donor–acceptor charge-transfer characteristics.^20^ Likewise, it was reported that PPy in PPy–TiO_2_ heterostructures acts not only as a photosensitizer but also as a connection bridge that broadens visible-light response and prolongs charge-carrier lifetime.^21^

Recent breakthroughs have underscored the immense promise of such hybrid photocatalysts. Studies on binary composites, including various PPy-based core–shell structures and heterojunctions (e.g., PPy@MoS_2_ and PPy/BiOBr), have demonstrated remarkable efficiencies and improved stability over traditional catalysts.^22–31^ For example, in water treatment applications, PPy-based composites with metal oxides like PPy@Zn_0_._5_Ni_0_._5_Fe_2_O_4_ have achieved 98.5% degradation of the organic pollutant malachite green dye,^32^ while PPy/BiOBr composites have shown nearly 100% removal of the heavy metal Cr­(VI) and rapid degradation of MB.^11^ The synergy with carbonaceous materials for contaminant removal is also well-documented; PPy/CN composites exhibited a 2-fold improvement in uranium reduction from water,^33^ and TiO_2_/CNT composites have proven highly effective for enhancing the photocatalytic degradation of pollutants due to the excellent electron-mediating role of CNTs.^19^

These studies clearly demonstrate that binary systems can substantially improve photocatalysis, but they also reveal a division of function. PPy–TiO_2_ systems primarily improve visible-light harvesting and interfacial charge separation, whereas PPy–CNT systems mainly provide efficient conductive pathways and enhanced carrier mobility. For instance, in PPy-SWCNT binary,^17^ π–π interaction between PPy and SWCNT was described as a conducting channel that facilitates high charge transportability, accelerates charge separation, and lowers charge-transfer resistance. Similarly, in BiOBr/PPy, PPy acted simultaneously as a photosensitizer and an electric conductor, enhancing both light absorption and e^–^/h^+^ transfer.^11^

Nevertheless, a persistent limitation across these reported hybrid systems (i.e., PPy-based binary composites/heterojunctions and carbon-assisted photocatalysts such as PPy/CN and TiO_2_/CNT) is incomplete charge separation and poor durability arising from weakly coupled or physically mixed interfaces; intensified e^–^/h^+^ recombination and interfacial degradation cumulatively undermine long-term performance.^14,34–37^ In PPy-containing photocatalysts, durability can be further compromised by polymer overoxidation, i.e., irreversible oxidative degradation of the PPy backbone beyond reversible p-doping, which disrupts the π-conjugated structure, lowers conductivity, and weakens interfacial charge-transport function during repeated operation. Because PPy is a p-type, π-conjugated semiconductor that can facilitate interfacial charge transfer and form junctions with n-type TiO_2_ to suppress recombination, durability and activity ultimately depend on engineering a continuously coupled interface rather than a simple physical mixture.^10,13^ In this context, ternary systems are not simply more complex composites but functionally integrated architectures designed to combine visible-light sensitization, semiconductor redox functionality, and fast conductive transport in a single coupled network.

Although the advantages of PPy as a photosensitizer and the roles of TiO_2_ and CNTs are well recognized, a comprehensive investigation of a PPy-centric ternary system remains limited. In particular, prior studies have rarely combined PPy, TiO_2_, and CNTs into a continuously coupled ternary heterojunction while simultaneously linking interfacial design to band alignment, reactive-species behavior, and degradation-pathway mapping under visible light. Thus, what remains insufficiently addressed is not only the fabrication of a PPy–TiO_2_–CNT ternary system itself but also the mechanistic integration of heterojunction design, charge-transfer behavior, ROS participation, and degradation-pathway verification within one visible-light photocatalytic framework. The ternary PPy–TiO_2_–CNT configuration is therefore pursued here to create a continuous electron-transport pathway between the visible-light-harvesting polymer and the oxide phase while improving charge separation, suppressing recombination, and enhancing operational durability beyond what is typically achieved in binary systems alone. To address this gap, this study develops a continuously coupled, mechanism-anchored PPy–TiO_2_–CNT heterojunction nanocomposite and integrates interface engineering with statistically guided process optimization to target band misalignment, weak interfacial coupling, and sluggish charge transport that accelerate e^–^/h^+^ recombination. The distinguishing aspect of the present work lies not merely in combining PPy, TiO_2_, and CNTs, but in the mechanism-guided construction and evaluation of a continuously coupled heterojunction, supported by correlated XPS, PL, DRS, EIS, scavenger, and LC–MS analyses. This integrated approach establishes a PPy-centric framework that suppresses recombination, promotes durability, and offers a mechanism-validated, low-cost route to practical visible-light photocatalysis for environmental remediation.

## 2 Experimental Section

### 2.1 Materials

All reagents were of analytical grade and used as received without further purification; deionized (DI) water and absolute ethanol were used throughout. Pyrrole (Py, ≥ 99%) was the monomer for the oxidative in situ polymerization step that produced the PPy phase coating of CNT/TiO_2_ during nanocomposite synthesis. TiO_2_ (anatase nanopowder) functioned as the semiconductor component of the heterostructure and as an inorganic scaffold for PPy growth. CNTs (produced by a chemical vapor deposition method) were supplied by Shenzhen Nanotechnologies Port Co. with diameters ranging from 40 to 60 nm and lengths ranging from 1 to 2 μm. Ferric chloride (FeCl_3_·6H_2_O, ACS grade) served as the oxidant (0.1 M aqueous) to initiate and sustain the oxidative polymerization of pyrrole via an in situ synthesis.

Nafion solution (≈5 wt % in lower alcohols) was used as the binder/ionomer to formulate the catalyst ink and to promote strong adhesion of the nanocomposite coating on substrates during electrode fabrication. Methylene blue (MB, dye standard, C_16_H_18_ClN_3_S·xH_2_O; Sigma-Aldrich), a powder, with dye content ≥ 82%, acted as the probe pollutant to evaluate photocatalytic activity under visible light; aqueous working solutions were freshly prepared before each experiment. Potassium nitrate (KNO_3_, ≥ 99%) served as the background electrolyte (0.01 M) for determining the point of zero charge (pH_p_zc) by the pH-drift (solid-addition) method. Sodium hydroxide (NaOH, 0.1 M) and hydrochloric acid (HCl, 0.1 M) were used to adjust solution pH in photocatalysis tests and for pH_p_zc measurements. All chemicals were purchased from commercial suppliers. Fluorine-doped tin oxide (FTO) glass substrates (1.5 cm × 4 cm; sheet resistance ∼7–15 Ω sq^–1^) served as conductive supports for doctor-blade deposition of the catalyst film to form working electrodes.

### 2.2 Methods

#### 2.2.1 Synthesis of Photocatalyst

The CNT-PPy-TiO_2_ nanocomposite photocatalyst was synthesized via an in situ polymerization method, as illustrated in Scheme 1. In a typical procedure, 6 mg of CNTs and 24 mg of TiO_2_ powder were dispersed in 10 mL of ethanol and magnetically stirred for 60 min to form a uniform suspension. The suspension was then ultrasonicated with a probe sonicator for 1 h to enhance dispersion and interfacial contact. Subsequently, 70 mg of the Py monomer was added dropwise to the suspension under continuous stirring, and the reaction was maintained for 1 h. Following this, 10 mL of an aqueous 0.1 M FeCl_3_ solution was slowly introduced as the oxidant, and the reaction mixture was stirred for 6 h at room temperature to facilitate the in situ polymerization of pyrrole onto the CNT-TiO_2_ composite.

**Scheme 1:**
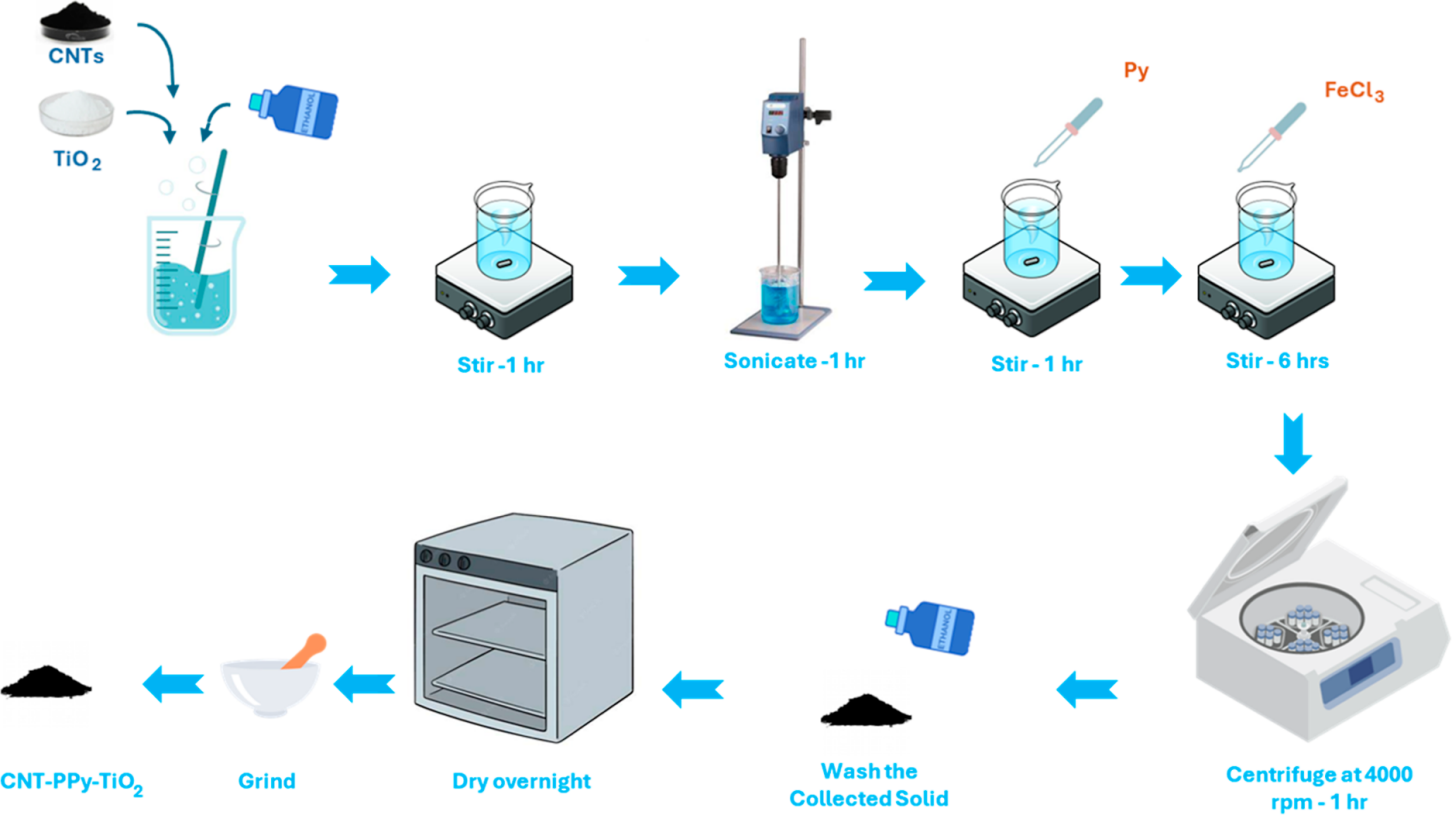
Schematic Illustration of the Preparation Procedure for CTPx Photocatalysts via in Situ Polymerization

After completion of the polymerization, the product was separated by centrifugation at 4000 rpm for 1 h as a single continuous centrifugation cycle. The resulting solid was washed thoroughly with distilled water and ethanol several times to remove unreacted monomers, oxidants, and byproducts. The purified material was dried overnight in an oven at 60 °C under air and then ground into a fine powder to obtain the CNT–PPy–TiO_2_ nanocomposite. The as-prepared catalyst was labeled CTP-3.

To prepare the other catalysts in the CTP series (CTP-1 to CTP-6), the same synthesis protocol described for CTP-3 was followed; only the precursor masses (i.e., CNT/TiO_2_:Py weight ratios) were adjusted. All catalysts (CTP-1-CTP-6) were therefore synthesized under identical conditions, with the compositions varied, as summarized in Table 1. For reproducibility, each composition was synthesized independently three times under the same preparation conditions, and the repeated samples showed no significant change in the photocatalytic degradation behavior or overall performance trend.

**Table 1:** Synthesized Nanocomposites PPy-TiO_2_–CNT (CTP-1 to CTP-6) Mass Ratios

nanocomposite	pyrrole (Py), mg	TiO_2_, mg	CNT, mg	total, mg
CTP-1	50	40	10	100
CTP-2	60	32	8	100
CTP-3	70	24	6	100
CTP-4	80	16	4	100
CTP-5	90	8	2	100
CTP-6	100	0	0	100

#### 2.2.2 Electrode Fabrication

The fabrication of the working electrode was carried out as follows. First, the as-prepared nanocomposite powder was dispersed in a mixture of 0.3 mL Nafion solution and 0.5 mL ethanol. The resulting suspension was thoroughly mixed and subsequently bath-sonicated for 10 min to ensure uniform dispersion. Meanwhile, the FTO glass substrate (1.5 cm × 4 cm) was cleaned by washing several times with deionized water and ethanol, followed by sequential sonication in water and then ethanol for 30 min each. After cleaning, the FTO substrate was dried in an oven at 60 °C overnight.

The homogeneous nanocomposite slurry containing Nafion and ethanol paste was deposited onto the conductive side of the cleaned FTO substrate using the doctor’s blade technique. A sharp knife was used to evenly spread the slurry across the substrate, ensuring uniform coating. The coated electrode was then left at room temperature for 6 h to allow for initial drying. Subsequently, the electrode was further dried in an oven at 60 °C for 12 h to remove residual solvent and binder, resulting in the final nanocomposite-coated FTO working electrode. The catalyst loading (mg cm^–2^) on the FTO substrate used for electrochemical measurements was determined according to eq 1

1 $$Catalyst\;load=\frac{mass\;of\;FTO\;with\;deposited\;catalyst-mass\;of\;bare\;FTO)}{coated\;FTO\;area}$$

On the basis of this calculation, the catalyst loading on the FTO substrate was approximately 0.3 mg cm^–2^.

#### 2.2.3 Characterization

The structural and morphological properties of the synthesized CNT-PPy-TiO_2_ nanocomposite photocatalysts were thoroughly investigated by using various characterization techniques. The surface morphology and local elemental composition of the prepared samples were examined by using a scanning electron microscope (SEM; Quanta 450 FEG, Czech Republic) combined with energy-dispersive X-ray spectroscopy (EDX/EDS) for local compositional analysis (20 mm, Oxford Instruments, UK). The formation of heterojunctions and nanoscale architecture was further examined by high-resolution transmission electron microscopy (HRTEM; Tecnai G2 20 S-Twin, FEI).

The crystalline phases present in the nanocomposites were identified using X-ray diffraction (XRD; Bruker D8, Cu Kα radiation, λ = 1.5406 Å, nickel filter) with a scanning range of 10° to 80°. Fourier transform infrared spectroscopy (FTIR; Bruker Tensor 27, 500–4000 cm^–1^) was utilized to determine the functional groups and confirm the successful incorporation of each component. The carbon structure and defects in the CNTs within the composite were evaluated by Raman spectroscopy (Renishaw in Via spectrometer, UK).

The elemental composition and chemical states of the constituents were further probed by X-ray photoelectron spectroscopy (XPS; Axis Ultra, Kratos Analytical, UK) using a monochromatic Al Kα source (1486.6 eV). The thermal stability of the nanocomposites was assessed via thermogravimetric analysis (TGA; SDT Q600, TA Instruments, USA). Liquid chromatography–mass spectrometry (LC–MS) was employed to identify photocatalytic degradation intermediates using a Waters Acquity UPLC system coupled to a Xevo TQ-S micro mass spectrometer and an Acquity UPLC BEH C18 (1.7 μm, 2.1 × 50 mm) column.

Electrochemical properties were investigated using an Autolab electrochemical workstation in a standard three-electrode configuration. The nanocomposite-coated graphite plate (30 mm × 15 mm) served as the working electrode, with a platinum rod as the counter electrode and an Ag/AgCl (3 M KCl) electrode as the reference. All measurements were conducted in 0.1 M KOH aqueous electrolyte. Linear sweep voltammetry (LSV) was performed at a scan rate of 20 mV/s, while electrochemical impedance spectroscopy (EIS) measurements were carried out over a frequency range of 0.1 Hz to 100 kHz with an AC perturbation amplitude of 10 mV.

Charge-carrier recombination behavior was evaluated by photoluminescence (PL) emission analysis (PL; PerkinElmer Lambda S55) using a Xe lamp at an excitation wavelength of 350 nm and a spectrofluorometer at 25 °C. The DRS analysis (PerkinElmer UV–Vis spectrophotometer, Lambda 35, USA) was conducted to obtain the reflectance spectra of the synthesized samples. For optical band gap estimation, the diffuse reflectance data were converted using the Kubelka–Munk function, *F*(*R*)=(1-*R*)^2^/2*R*, where *R* is the measured reflectance. The Tauc plots were then constructed by plotting [*F*(*R*)*h*ν]^1/2^versus *h*ν, assuming an indirect allowed electronic transition. Here, *h* is Planck’s constant and ν is the photon frequency, while the exponent 1/2 was selected for the indirect transition model. The optical band gap (*E*_g_)­was determined by linear extrapolation of the absorption-edge region to the energy axis.

#### 2.2.4 Evaluation of Photocatalytic Activity

Photocatalytic activity was evaluated using MB as a model pollutant under visible-light irradiation. In a typical experiment, 50 mg of the photocatalyst was dispersed in 100 mL of MB solution (20 mg·L^–1^), corresponding to a catalyst dosage of 0.5 mg mL^–1^, in a glass reactor and magnetically stirred. Before illumination, the suspension was kept in the dark for 60 min to establish adsorption–desorption equilibrium. Illumination was then provided by a visible-light lamp equipped with a long-pass cutoff filter (λ > 420 nm). The distance between the lamp and the reactor surface was fixed at 10 cm in all photodegradation experiments. The light intensity at the reactor surface was 90 mW cm^–2^.

At fixed intervals of 15 min, 3 mL aliquots were withdrawn and immediately centrifuged to remove suspended solids. The supernatant was analyzed by UV–Vis spectrophotometry, monitoring the MB absorbance at 664 nm. Concentration changes were calculated assuming Beer–Lambert proportionality: *C*_t_/*C*_o_ = *A*_t_/*A*_o_, where Co and Ao are the concentration and absorbance at *t* = 0 (after dark equilibration), and *C*_t_ and *A*_t_ are the corresponding values at irradiation time *t*. The degradation efficiency was computed by eq 2.

2 $$Degradation\;efficiency\;\%=\left(1-\frac{C_{t}}{C_{o}}\right)x100$$

Kinetic analysis followed a pseudo-first-order model (eq 3) by linear regression of ln­(*C*_t_/*C*_o_) versus time

3 $$-\ln\left(\frac{C_{t}}{C_{o}}\right)=k.t$$

where *k* is the apparent rate constant (reported in min^–1^ when time is in minutes). Unless otherwise specified for parametric studies (e.g., catalyst dosage, initial MB concentration, or solution pH), all tests were conducted under identical conditions for consistent comparison. All photocatalytic experiments were independently repeated three times under identical conditions, and the reported data are presented as mean ± standard deviation (SD).

#### 2.2.5 Point of Zero Charge (PZC)

The zero charge point pH (pH_p_zc) corresponds to the pH value for which the net charge of the surface of the adsorbent is zero. The solid addition method was used to determine the zero-point charge of the CTP-3 nanocomposite material (50 mg) was added into each of five beakers containing 50 mL of KNO_3_ solution (0.01 M). The initial pH of the KNO_3_ solutions was adjusted to pH 3, 5, 7, 9, and 11 using NaOH (0.1 M) and HCl (0.1 M) solutions. After 24 h of agitation, all beakers were withdrawn from the stirrer, and the final pH was measured using a typical pH meter. The intersection points on the curve (pH*_i_*–pHf) versus pH*_i_* were estimated as the pH value of zero charge point.

#### 2.2.6 Reaction Pathway Experiment

The reaction pathway for MB photodegradation was probed for the optimized PPy–TiO_2_–CNT photocatalyst (CTP-3) under the same visible-light conditions used for kinetics (lamp/reactor details in Section 2.2.4). At *t* = 0, 15, 30, 45, and 60 min, 2 mL aliquots were withdrawn and immediately quenched by excluding light (wrapped in foil), then syringe-filtered (0.22 μm PTFE). Samples were labeled MB0–MB4 in chronological order. Processed aliquots were analyzed by liquid chromatography–mass spectrometry (LC–MS) using a reversed-phase C18 column with a water/acetonitrile gradient (0.1% formic acid, v/v), electrospray ionization in positive mode (ESI^+^), and full-scan acquisition over *m*/*z* 50–600. Data processing included baseline correction, centroiding, and extraction of ion traces for the parent ion (*m*/*z* ≈ 284) and characteristic fragment clusters. Dark and photolysis controls were prepared and handled identically to verify that observed transformations were photocatalytic.

## 3 Results and Discussion

### 3.1 X-ray Diffraction (XRD)

The XRD patterns of the PPy–TiO_2_–CNT nanocomposites (Figure 1) confirm the coexistence of polymer, oxide, and carbonaceous phases with strong interfacial interactions. The diffractogram shows distinct peaks at 2θ ≈ 25.3°, 37.8°, 48.0°, 54.0°,55.1°, and 62.7°, which are indexed to the (101), (004), (200), (105), (211), and (204) planes of anatase TiO_2_ (JCPDS No. 21–1272). Additional weaker reflections at higher diffraction angles can also be assigned to anatase TiO_2_, confirming that anatase remained the dominant crystalline phase throughout the composite series. The dominance of the (101) reflection is typical of anatase-phase nanocrystals, consistent with previous studies where TiO_2_ was incorporated in PPy-based heterostructures.^38,39^ The absence of rutile reflections suggests that the polymerization and CNT incorporation suppressed phase transformation, a stabilizing effect also noted in PPy/TiO_2_ and PPy/oxide composites.^39,40^ Moreover, no impurity-related reflections were detected, indicating that no secondary crystalline phases formed during synthesis.

**Figure 1:**
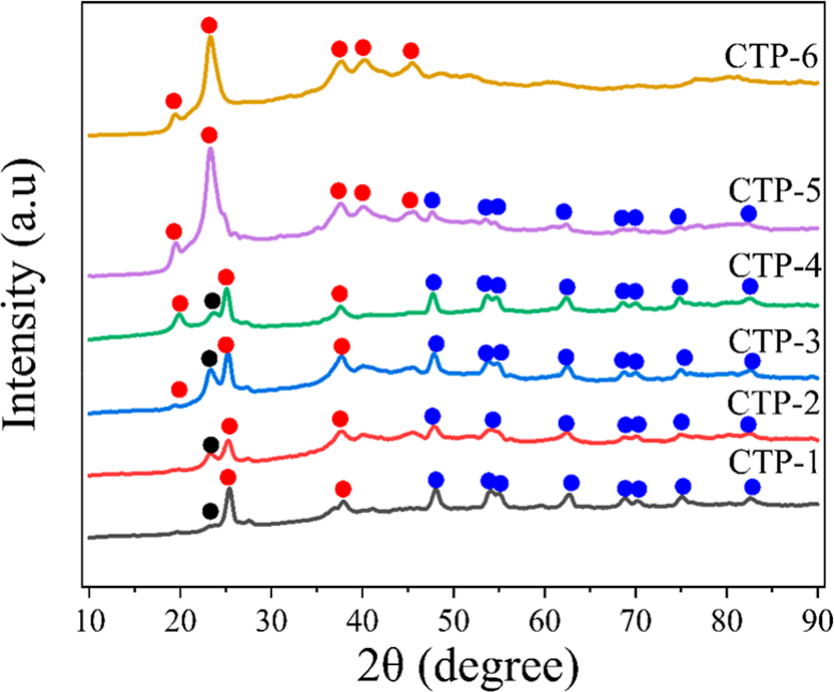
X-ray diffraction (XRD) patterns of the PPy–TiO_2_–CNT nanocomposite series (CTP-1 to CTP-6).

A broad diffraction feature around 2θ ≈ 24–26° is observed, corresponding to the (002) plane of CNTs and the amorphous halo of PPy. In PPy/CNT systems, this broadening is generally attributed to π–π conjugation between the polymer chains and graphitic carbon, which enhances charge transfer and electronic conductivity.^38^ The overlap of this band with TiO_2_ (101) reflection indicates strong hybridization at the interface. Compared to pristine TiO_2_, the nanocomposites exhibit a reduction in peak intensity and slight broadening, suggesting decreased crystallite size and partial amorphization due to the PPy coating. Similar reductions have been reported in PPy/ZnO and PPy/Cu_2_O composites, where polymer layers suppressed crystal growth and improved interfacial stability.^38,39^ To further support this observation quantitatively, the crystallite size of the TiO_2_ phase was estimated from the anatase (101) peak using the Scherrer equation, as given in eq 4.

4 $$D=\frac{0.9\lambda }{\beta \cos\left(\theta \right)}$$

where *D* is the crystallite size, *K* is the shape factor (0.9), λ is the X-ray wavelength of Cu Kα radiation (0.15406 nm), β is the full width at half-maximum (fwhm, in radians), and θ is the Bragg angle. The calculated crystallite sizes are summarized in Table S1. As shown in Table S1, the estimated crystallite sizes for CTP-1 to CTP-4 were in the range of 9.44–11.14 nm, confirming that the TiO_2_ domains remained in the nanocrystalline regime after composite formation. This result is consistent with the observed peak broadening and supports the formation of intimate interfacial contact among PPy, TiO_2_, and CNT. For CTP-5, however, no reliable crystallite size was reported because the fitted low-angle feature was centered at approximately 2θ = 23.4°, rather than near 25.3°, as expected for the characteristic anatase TiO_2_ (101) reflection. This pronounced shift indicates that the feature cannot be confidently assigned to anatase TiO_2_ and is more reasonably attributed to the overlapping broad PPy/CNT-related contribution in the low-angle region. Overall, the Scherrer analysis provides quantitative support for the structural interpretation derived from the XRD patterns and further strengthens the evidence for nanoscale TiO_2_ domains within the PPy-TiO_2_–CNT heterostructures.

The absence of additional impurity peaks confirms that no secondary crystalline phases were formed during synthesis. This is consistent with reports where in situ polymerization of PPy onto oxide/carbon substrates yielded clean hybrid patterns without extraneous reflections.^39,41^

### 3.2 Raman Spectroscopy

The Raman spectra of the PPy–TiO_2_–CNT nanocomposites (Figure 2) provide clear evidence of the coexistence of the polymer, oxide, and carbon phases, as well as strong interfacial interactions. The characteristic vibrational modes of anatase TiO_2_ are observed at ∼144 cm^–1^ (*E*_g_), 399 cm^–1^ (B_1g_), 513 cm^–1^ (A_1g_), and 639 cm^–1^ (*E*_g_), in agreement with standard assignments for the anatase phase.^42^ The strong intensity of the 144 cm^–1^ mode confirms the dominance of the anatase phase, consistent with XRD results. Compared to bare TiO_2_, these modes show slight broadening and red-shifting, suggesting interfacial charge transfer between TiO_2_ and PPy, which is often observed in PPy/oxide nanocomposites.^43^

**Figure 2:**
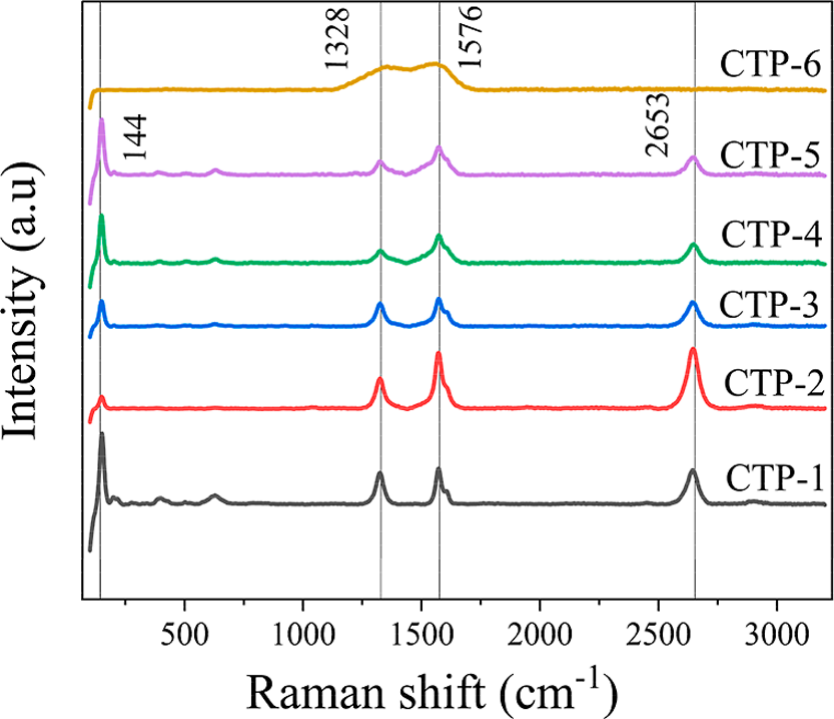
Raman spectra of the PPy–TiO_2_–CNT nanocomposite series (CTP-1 to CTP-6).

The PPy contribution is evidenced by bands in the range of 930–980 cm^–1^ (C–H in-plane deformation), ∼1050–1080 cm^–1^ (N–H in-plane bending), and ∼1330–1580 cm^–1^ corresponding to the C–C and C=C stretching vibrations of benzenoid and quinoid structures in the PPy backbone.^44,45^ The shift and broadening of these bands compared to pristine PPy indicate π–π stacking interactions with CNTs and possible doping-induced structural modifications. CNT incorporation is reflected by the D and G bands at ∼1345 cm^–1^ and ∼1580 cm^–1^, respectively. The D band corresponds to disorder-induced vibrations, while the G band arises from graphitic C=C stretching.^46^ The intensity ratio (ID/IG) for the composite is higher than that of pristine CNTs, indicating enhanced structural disorder due to polymer wrapping and interfacial hybridization with TiO_2_. Such modifications have been linked to improved charge carrier mobility in similar PPy/CNT systems.^47^

Notably, the overlap of the PPy quinoid (∼1580 cm^–1^) with the CNT G band results in band broadening, reflecting strong electronic coupling.^45,47^ Taken together, the Raman results provide more than simple phase confirmation. The band broadening and shifts associated with PPy, TiO_2_, and CNT indicate electronic interaction among the three components, while the modified CNT D/G response and the overlap of the PPy quinoid band with the CNT G band are consistent with closer polymer–carbon coupling. Accordingly, the Raman data support the presence of an electronically interacting PPy–TiO_2_–CNT network, which is consistent with the proposed continuously coupled heterojunction rather than a simple physical mixture of individual components. These spectral signatures corroborate the formation of a strongly coupled heterojunction, which is beneficial for suppressing recombination and enhancing photocatalytic activity.^43,44^

### 3.3 Fourier Transform Infrared (FTIR)

The FTIR spectra of the PPy–TiO_2_–CNT nanocomposites (Figure 3) confirm the coexistence and interaction of all components. The characteristic absorption of PPy is evident from the stretching vibrations at ∼1540 cm^–1^ and ∼1470 cm^–1^, corresponding to quinoid and benzenoid ring modes, respectively. These bands confirm the successful polymerization of pyrrole into the conjugated backbone.^48^ Additionally, peaks at ∼1290–1310 cm^–1^ and ∼1040–1060 cm^–1^ are attributed to C–N stretching of pyrrole rings and in-plane deformation of C–H bonds, which are typical features of PPy.^48^ The spectrum also exhibits distinct bands associated with TiO_2_.

**Figure 3:**
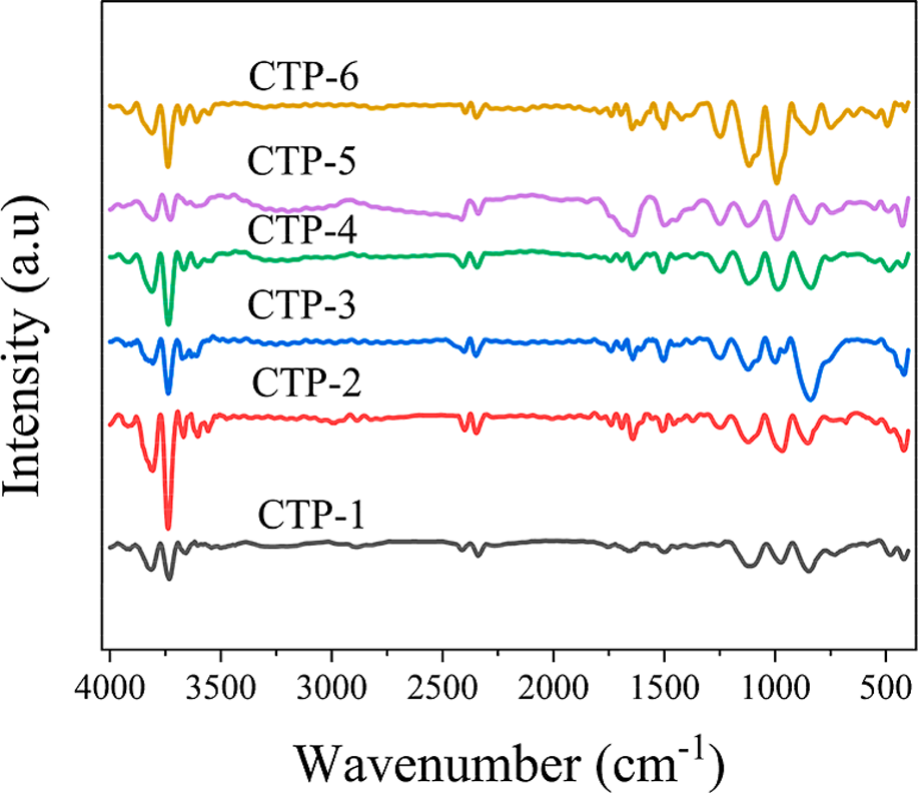
FTIR spectra of the PPy–TiO_2_–CNT nanocomposite series (CTP-1 to CTP-6).

The broad absorption below 700 cm^–1^ is assigned to Ti–O–Ti stretching vibrations, confirming the presence of anatase TiO_2_ in the hybrid.^49^ The intensity reduction and slight red shift of this band compared to bare TiO_2_ indicate strong interfacial interactions between PPy and TiO_2_, which can facilitate charge transfer and electron–hole separation, as reported in PPy/TiO_2_ composites.^50,51^ CNT incorporation is further evidenced by skeletal vibrations in the 1550–1600 cm^–1^ region, corresponding to C=C stretching of graphitic domains.^48^ These overlap with PPy quinoid vibrations but contribute to the broadening of the peak, reflecting π–π conjugation between CNTs and PPy chains. This interaction has been reported to improve electron delocalization and enhance stability in PPy/CNT nanostructures.^52^

A broad absorption band around 3400 cm^–1^ corresponds to O–H stretching from adsorbed water or surface hydroxyls. This feature is commonly observed in polymer/oxide systems and contributes to hydrophilicity and interfacial hydrogen bonding.^50^ Minor peaks in the 1640–1660 cm^–1^ range can also be attributed to C=O stretching of surface oxygen species, indicating possible partial oxidation of PPy during synthesis.^48^ Overall, the FTIR spectrum confirms not only the coexistence of PPy, TiO_2_, and CNT, but also their interfacial interaction. In particular, the shifted Ti–O–Ti and PPy-related bands, together with the broadened graphitic C=C region, are consistent with polymer–oxide and polymer-CNT coupling. These spectral changes support the formation of interfacial contact pathways that can facilitate charge transfer across the hybrid structure, thereby complementing the Raman, XPS, and electrochemical evidence for a continuously coupled heterojunction.

### 3.4 X-ray Photoelectron Spectroscopy (XPS)

The elemental surface chemical states and compositions of PPy, TiO_2_, and the PPy–TiO_2_–CNT composite (CTP-3) were analyzed by XPS (Figure S1). The XPS survey (wide-scan) spectrum (Figure S1a) is a broad binding-energy scan used to identify the elements present on the sample surface and their overall atomic contributions. In this spectrum, clear signals from C 1s, N 1s, O 1s, and Ti 2p are observed, confirming the coexistence of PPy/CNT (C, N) with TiO_2_ (Ti, O) in the composite.^11,53^ The fitted binding energies, fwhm values, and relative peak areas obtained from the Shirley-background deconvolution of the high-resolution spectra are summarized in Table S2. The weak, high-binding-energy oxygen contribution typically arises from adsorbed H_2_O/CO_2_ accumulated during sample handling.^11^ The C 1s region (Figure S1b) is dominated by a peak at 284.58 eV attributable to C–C/C=C (graphitic sp^2^ carbon from CNT and the PPy backbone), accompanied by a shoulder at ∼285.7–286.3 eV assigned to C–N/C–O species from PPy and surface oxidation. A very weak component may appear near ∼288–289 eV (C=O/N–C=O) and a π–π* shakeup around ∼290–291 eV, consistent with conjugated carbon frameworks.^11,17^ These features verify the presence of PPy and CNT and indicate interfacial bonding/functional groups at the oxide surface.

The N 1s spectrum (Figure S1c) is represented by a broad envelope centered at 399.67 eV (Table S2), which is consistent with nitrogen species in the PPy backbone. Because only one broad component was reliably fitted, the present spectrum confirms PPy-derived nitrogen on the composite surface but does not support strong quantitative separation of neutral, polaronic, and bipolaronic nitrogen species.^11,17^ In the Ti 2p region (Figure S1d), the fitted components at 458.72 and 464.44 eV (Table S2) correspond to Ti 2p_3_/_2_ and Ti 2p_1_/_2_, respectively, with a spin–orbit splitting of approximately 5.72 eV, which is characteristic of Ti^4+^ in TiO_2_. These fitted values therefore support Ti^4+^ as the dominant titanium oxidation state in the composite.^10,21^ Similarly, the O 1s spectrum (Figure S1e) was fitted with two components after Shirley-background subtraction. The main peak at 529.98 eV is assigned to lattice oxygen (Ti–O) in TiO_2_, while the broader component at 531.09 eV is reasonably attributed to surface hydroxyl groups and/or adsorbed oxygenated species.^10,11^ The larger relative area of the lower-binding-energy component as summarized in Table S2 indicates that lattice oxygen is dominant, whereas the higher-binding-energy contribution suggests the presence of surface oxygen species that may influence adsorption and photocatalytic interfacial processes.

Overall, the XPS results confirm the successful surface assembly of PPy/CNT and TiO_2_ in the CTP-3 composite. The C 1s and O 1s features are consistent with conjugated carbon, C–N/C–O functionalities, lattice oxygen, and surface hydroxyl/adsorbed oxygen species, while the Ti 2p doublet confirms Ti^4+^ as the dominant titanium state. At the same time, the revised deconvolution indicates that the present XPS data should be interpreted conservatively: they support interfacial functionalization and surface electronic interaction but do not by themselves fully define the spatial arrangement of the interface. Therefore, the conclusion of a continuously coupled heterojunction is drawn from the combined evidence of XPS together with SEM/TEM, Raman/FTIR shifts, PL quenching, reduced EIS charge-transfer resistance, and VB-XPS-supported band analysis, rather than from XPS alone. These surface/electronic signatures align with the observed photocatalytic behavior of CTP-3, where enhanced interfacial charge transport and abundant hydroxylated sites promote electron-driven O_2_ → H_2_O_2_→^•^OH pathways for MB oxidation while direct hole-to-^•^OH formation is disfavored.^10,11^

### 3.5 Thermogravimetric Analysis (TGA)

The thermal stability of the CTP nanocomposites was evaluated by thermogravimetric analysis (TGA) in air at a heating rate of 10 °C min^–1^ to examine the degradation behavior of pristine PPy (CTP-6) and the PPy–TiO_2_–CNT composite series (CTP-1 to CTP-5), as well as the influence of TiO_2_ and CNT incorporation on the decomposition profile and residual mass (Figure S2a,b). The three degradation stages were identified from the TG curves by defining the onset and end of each mass-loss region and confirming the corresponding DTG extrema. The mass loss associated with each stage was then calculated from the difference in sample mass between the beginning and end of the selected temperature interval. The TG/DTG profiles show a clear three-step decomposition pattern. Region I (30–120 °C) exhibits a mass loss of approximately 3–5%, with DTG minima centered at 60–70 °C, and is attributed to the removal of physisorbed water and trapped volatile species. This behavior is commonly observed in PPy/oxide hybrid systems due to moisture retention associated with surface porosity and polar functional groups.^53^ Region II (150–450 °C) represents the major decomposition step, with derivative maxima between 413 and 443 °C and an overall mass loss of ∼45–55%. This step is attributed to oxidative decomposition of the PPy backbone.^20,53^

Compared with pristine PPy (CTP-6), the composite samples display a sharper and more gradual mass-loss behavior in Region II, indicating that the presence of TiO_2_ and CNTs modifies the thermal degradation pathway of PPy. This behavior suggests stronger interfacial interaction among PPy, TiO_2_, and CNT, which can restrict polymer-chain mobility and delay rapid oxidative scission. Such stabilization is consistent with the formation of an integrated hybrid structure rather than a simple physical mixture.^20^ Region III (500–650 °C) corresponds to ∼15–25% weight loss, with derivative peaks centered at 600–626 °C, assigned to the oxidative combustion of CNTs and the phase transformation of TiO_2_ from anatase to rutile. It is noteworthy that TiO_2_ is thermal stable (remaining stable above 1000 °C), but it may undergo a phase transformation from anatase to rutile at approximately 600 to 700 °C.^54^ Above 650 °C, the TG curves approach a near-plateau, with residual masses at 800 °C of 23.6 wt % (CTP-1), 31.7 wt % (CTP-2), 36.8 wt % (CTP-3), 34.9 wt % (CTP-4), 33.1 wt % (CTP-5), and 7.7 wt % (CTP-6). For the optimal sample, CTP-3, the measured residual mass at 800 °C is 36.8 wt %, which is notably higher than the nominal TiO_2_ fraction of 24 wt % reported in Table 1. This difference indicates that the final residue cannot be attributed to TiO_2_ alone. Rather, it should be interpreted as an apparent inorganic-rich residue that may also include oxidized carbonaceous remnants formed during multistep decomposition in air. Accordingly, the residual mass at 800 °C provides only semiquantitative evidence of inorganic content and relative differences in polymer/inorganic balance among the samples, rather than definitive confirmation of exact precursor-ratio retention after in situ polymerization.

These final residues represent the apparent nonvolatile fraction of each sample and are consistent with increasing inorganic TiO_2_-containing content in the composite samples relative to neat PPy (CTP-6). However, the residue at 800 °C should be interpreted as an apparent inorganic-rich residue rather than pure TiO_2_ alone because overlapping PPy/CNT decomposition and residual oxidized carbonaceous species may also contribute to the final plateau. Therefore, the TGA results provide semiquantitative support for the successful incorporation of oxide-containing inorganic content into the CTP series and for differences in polymer/inorganic balance among the samples, but they should not be taken as definitive proof that the exact intended precursor ratios were retained after in situ polymerization. Overall, the TGA results confirm that incorporation of TiO_2_ and CNTs improves the thermal stability of the PPy matrix, modifies the degradation pathway, and increases the final inorganic-rich residue, in agreement with the SEM/TEM, FTIR, Raman, and XPS results that support successful formation of the PPy–TiO_2_–CNT heterostructure.

### 3.6 Morphological Characterization and Surface Properties (SEM and TEM)

The SEM micrographs (Figure 4) reveal systematic morphology changes with composition. Pristine PPy (CTP-6; panels a,b) shows a compact, cauliflower-like surface composed of coalesced globular domains on the order of (∼500 nm −2 μm), indicating limited porosity and tortuous charge-transport pathways. Introducing TiO_2_ and CNTs (CTP-1 and CTP-2; panels c–f) disrupts the large PPy agglomerates and produces a more open, heterogeneous network in which TiO_2_ nanoparticles decorate and bridge the polymer matrix while CNTs form filamentary backbones, improving dispersion and interparticle connectivity. At intermediate compositions (CTP-3 and CTP-4; panels g–j) the microstructure appears more evenly distributed than in pristine PPy, with finer hybrid aggregates spread across the surface and with closer apparent mixing of the polymer, oxide, and carbon components. Rather than assigning specific porosity features from SEM alone, these images are interpreted more conservatively as evidence of improved dispersion and interfacial contact relative to PPy-rich domains. At higher PPy loadings (CTP-5; panels k,l) partial polymer overgrowth re-emerges, locally burying CNTs/TiO_2_ and yielding a denser surface relative to CTP-3 and CTP-4.

**Figure 4:**
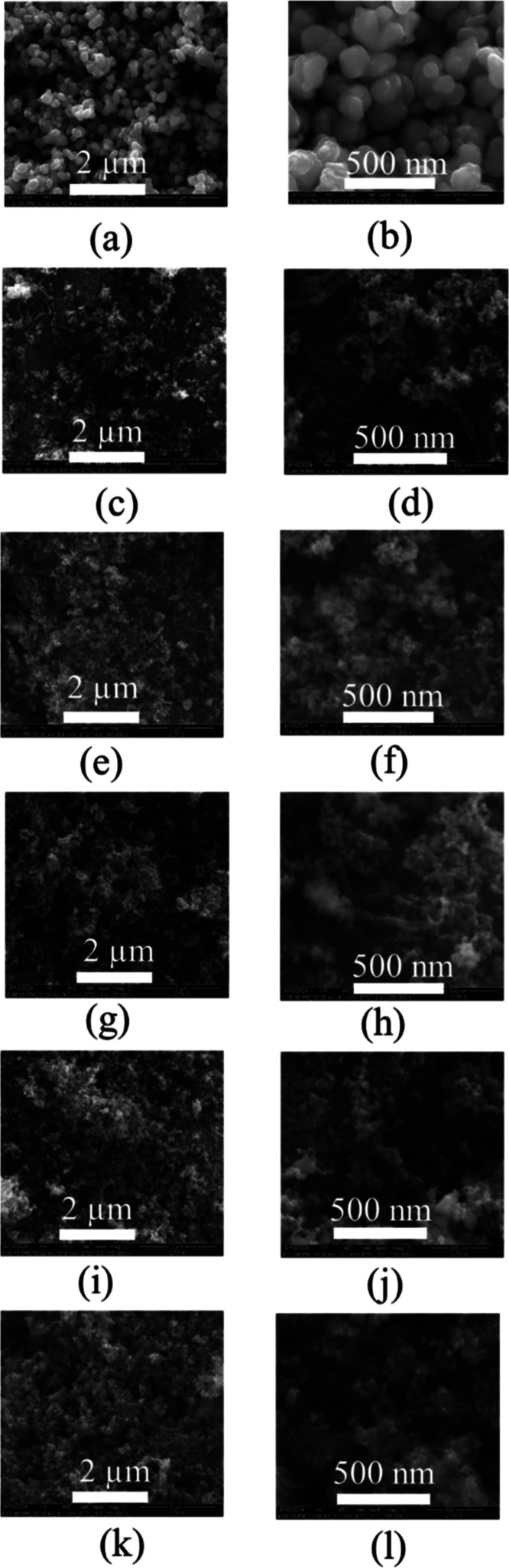
SEM micrographs of CNT–TiO_2_–PPy nanocomposites (CTPx). Panels are arranged in two columns by magnification: left column—50,000× (field of view ≈ 2 μm); right column—200,000× (field of view ≈ 500 nm). Rows correspond to samples: (a,b) PPy (CTP-6); (c,d) CTP-1; (e,f) CTP-2; (g,h) CTP-3; (i,j) CTP-4; and (k,l) CTP-5.

Figure S3 collectively illustrates the nanoscale association among PPy, TiO_2_, and CNT in the hybrid samples. In PPy alone (panel f), the polymer forms amorphous, cauliflower-like globules that coalesce into dense aggregates with tortuous charge-transport pathways, which is qualitatively consistent with the stronger PL response and larger Nyquist semicircle observed for the PPy-rich sample. Upon hybridization with CNTs and TiO_2_ (panels a–e), PPy is observed in closer association with CNT sidewalls and embeds TiO_2_ nanocrystals, indicating the formation of polymer–oxide–carbon contact regions rather than isolated PPy globules. These SEM/TEM observations are discussed here as qualitative structural evidence of PPy–TiO_2_–CNT hybrid integration and interfacial contact; no rigorous porosity or feature-size statistics are inferred from the present images.

For the optimized CTP-3 sample, this more integrated morphology is consistent with a percolated PPy/CNT pathway together with closer PPy–TiO_2_ contact, both of which would favor electron extraction and shorten interfacial charge-transfer distances. Thus, the morphology results support the proposed continuously coupled heterojunction at the structural level and are in good agreement with the Raman/FTIR shifts, the PL quenching behavior, and the reduced EIS charge-transfer resistance discussed in the subsequent sections.

### 3.7 Optical Properties and Charge-Carrier Behavior

Photoluminescence (PL) was used to gauge charge separation in the materials. Lower PL means fewer electron–hole pairs recombine and more charges remain available for reactions.^55,56^ Pristine PPy shows a main band at ∼516 nm with a weak shoulder near ∼386 nm and faint red-tail features (∼625–753 nm), consistent with the backbone and polaron/bipolaron emissions (Figure 5a).

**Figure 5:**
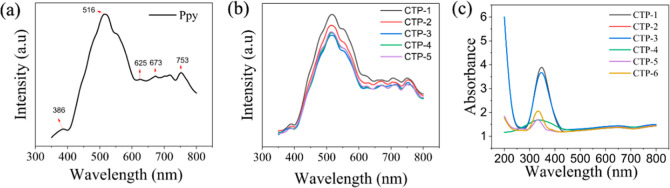
(a) Photoluminescence spectrum of pristine PPy; (b) Photoluminescence spectrum of PPy–TiO_2_–CNT nanocomposites (CTP-1 to CTP-5) and (c) UV–Vis absorption spectra of CTP-1 to CTP-6.

In the composites (CTP-1-CTP-5), the emission maximum remains at 514–518 nm with a similar near-UV shoulder, but the intensity is progressively quenched, with the strongest quenching observed for CTP-3, indicating more effective interfacial charge transfer and weaker recombination (Figure 5b).^57^ Complementing these findings, UV–vis spectroscopy (Figure 5c) and Tauc analysis were used to determine the indirect band gaps (Eg) of the materials, which were found to be ∼2.70 eV (CTP-1), 2.65 eV (CTP-2), 2.10 eV (CTP-3), 1.85 eV (CTP-4), 1.55 eV (CTP-5), and 2.60 eV (CTP-6), as shown in Figure S4a–g. An effective photocatalyst must strike a balance between a narrow band gap for enhanced visible-light harvesting and a sufficiently large band gap to maintain the redox potential required to generate reactive oxygen species like ^•^O_2_^–^ and ^•^OH.^58^

The results indicate that the CTP-3 sample achieves the most favorable balance, as its absorption edges extend well into the visible spectrum (∼590–670 nm), enabling strong photon capture while likely preserving the necessary driving force for catalysis. In contrast, the wider band gaps of CTP-1, CTP-2, and CTP-6 limit their visible-light response, while the very narrow band gap of CTP-5, despite its broad absorption, likely suffers from insufficient band-edge potential and may promote recombination at defect states.

By integration of the PL, DRS/Tauc, and EIS results, the superior behavior of CTP-3 can be interpreted in interfacial terms rather than only as a performance ranking. The strongest PL quenching indicates the most effective suppression of radiative e^–^/h^+^ recombination,^59^ the balanced optical gap and extended visible-light response favor photon utilization under visible irradiation,^60^ and the smallest Nyquist semicircle indicates the lowest interfacial charge-transfer resistance.^61^ In agreement, the Nyquist plots display the smallest semicircle for CTP-3 (Figure S5), evidencing lower interfacial resistance and faster charge transfer than the other samples. Together, these results suggest that CTP-3 possesses the most effective electronic communication among PPy, TiO_2_, and CNTs, consistent with a more continuously coupled heterojunction that facilitates carrier separation and transport under visible light.

### 3.8 Photocatalytic Performance Screening and Selection

To identify the most effective photocatalyst, the photocatalytic activities of all six CNT-PPy-TiO_2_ nanocomposites (CTP-1 to CTP-6) were systematically evaluated for the degradation of MB under visible light irradiation. Each photocatalyst was tested under identical conditions, with an initial MB concentration of 20 mg L^–1^, a catalyst dosage of 0.5 mg mL^–1^, and an irradiation period of 150 min, using a 500 W xenon lamp as the light source. The degradation efficiency was assessed by monitoring the decrease in absorbance at 664 nm using UV–vis spectroscopy. Prior to illumination, the suspensions were maintained in the dark to establish adsorption–desorption equilibrium, ensuring that subsequent concentration changes predominantly reflect photocatalytic reactions rather than initial adsorption. For each catalyst, the degradation experiment was repeated three times independently (*n* = 3), and the plotted values are presented as mean ± SD.

Figure 6 summarizes the screening outcomes. In Figure 6a, the photolysis control exhibits negligible MB removal, confirming that direct dye photobleaching under the lamp is minimal and that the observed concentration decay is catalyst-driven. Upon visible-light exposure, the composites show clearly differentiated degradation profiles, where CTP-3 displays the most rapid decline in *C*_t_/*C*_0_, followed by CTP-2 ≈ CTP-1, then CTP-4 and CTP-5. In contrast, CTP-6 shows only a marginal reduction in *C*_t_/*C*_0_, indicating weak activity under the same conditions. Consistently, the pseudo-first-order representations in Figure 6b (−ln­(*C*_t_/*C*_0_) vs time) show the steepest slope for CTP-3, evidencing the fastest apparent degradation kinetics among the series.

**Figure 6:**
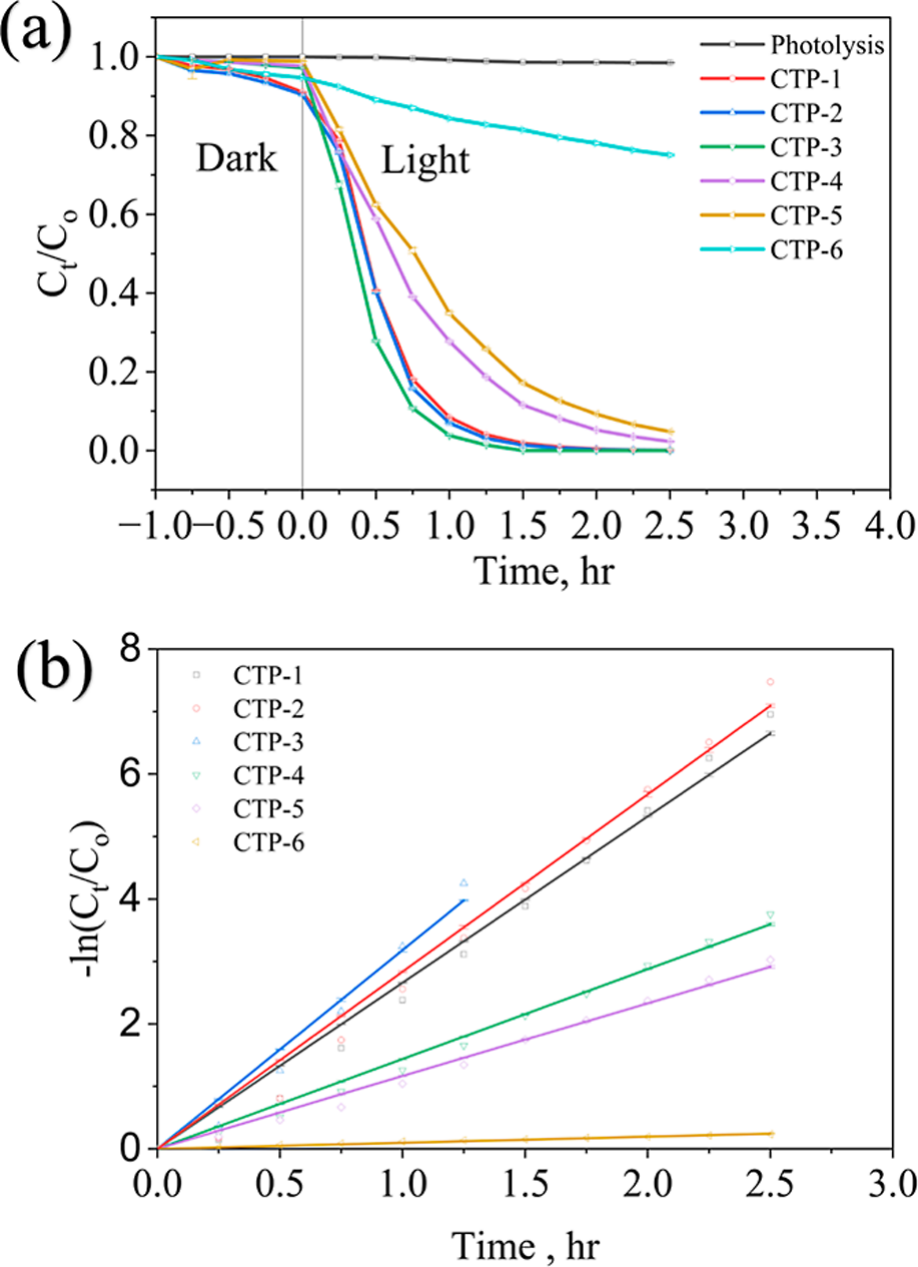
MB degradation over PPy–TiO_2_–CNT photocatalysts (CTP-1-CTP-6): (a) *C*_t_/*C*_0_ vs time and (b) pseudo-first-order kinetic plots, −ln­(*C*_t_/*C*_0_) vs time.

To quantify these trends, the kinetic parameters extracted from the pseudo-first-order model (−ln­(*C*_t_/*C*_0_) = kt) are compiled in Table 2. All catalysts show high linearity (*R* ^2^ = 0.94–0.995), supporting the suitability of the pseudo-first-order approximation within the tested time window. Importantly, the kinetic ranking is governed by the rate constant k (slope), and CTP-3 achieves the highest value (*k* = 0.0531 min^–1^), confirming its kinetic superiority. Although CTP-1 and CTP-2 present slightly higher *R* ^2^, CTP-3 maintains the steepest kinetic slope, indicating more rapid MB conversion under visible light. The weak performance of CTP-6 (*k* = 0.0016 min^–1^) further indicates that PPy alone is insufficient to drive efficient photodegradation under these conditions, emphasizing that the enhanced activity arises from the composite architecture and synergistic charge-handling pathways enabled by TiO_2_ and CNT.

**Table 2:** Pseudo-first-order Kinetic Parameters for MB Photodegradation Over PPy–TiO_2_–CNT Photocatalysts, Reported as Mean ± SD

catalyst	k (mean ± SD)		*R* ^2^
	hr^–1^	min^–1^	
CTP-1	2.660 ± 0.063	0.0443 ± 0.0011	0.9944
CTP-2	2.837 ± 0.068	0.0473 ± 0.0011	0.9944
CTP-3	3.182 ± 0.131	0.0530 ± 0.0022	0.9917
CTP-4	1.439 ± 0.026	0.0240 ± 0.0004	0.9966
CTP-5	1.166 ± 0.023	0.0194 ± 0.0004	0.9962
CTP-6	0.098 ± 0.002	0.0016 ± 0.00003	0.9963

To further place the present results in context, a comparison with representative binary PPy–TiO_2_ and PPy–CNT photocatalysts reported for organic pollutant degradation is provided in Table S3. Although direct cross-study comparison should be interpreted cautiously because pollutant type, light source, catalyst dosage, pH, reaction time, and kinetic model vary among reports, the optimized CTP-3 photocatalyst in the present work (*k* = 0.0531 min^–1^ for methylene blue degradation) shows kinetically competitive performance relative to representative binary PPy–TiO_2_ systems reported in the literature. For example, a PPy/TiO_2_ binary photocatalyst was reported to exhibit a rate constant of 2.19 × 10^–2^ min^–1^ for methylene blue degradation under visible light, which is lower than that obtained for CTP-3 under the present conditions.^62^ Other binary PPy–TiO_2_ systems have also shown high degradation efficiencies, such as 93% methylene blue removal in 90 min under optimized conditions.^63^ In addition, binary PPy–CNT systems reported for pollutant degradation also exhibit substantial activity, including 78.30% methylene blue degradation in 35 min, further supporting the beneficial role of conductive carbon nanotube coupling in PPy-based photocatalysts.^64^

This literature comparison, together with the very low activity of PPy alone in the present work (CTP-6, *k* = 0.0016 min^–1^), indicates that the enhanced visible-light photocatalytic performance cannot be attributed to PPy alone. Rather, the improved activity is reasonably ascribed to the synergistic effects of the ternary PPy–TiO_2_–CNT architecture, in which TiO_2_ provides the photocatalytically active semiconductor framework, PPy extends visible-light absorption, and CNT facilitates interfacial charge separation and electron transport, thereby improving the utilization of photoexcited charge carriers.

Consistent with the activity trends, three independent characterization readouts singled out CTP-3: (i) PL shows the strongest quenching for CTP-3, indicating the most effective suppression of e^–^/h^+^ recombination; (ii) DRS/Tauc reveals the most extended visible-light response and the smallest optical band gap (≈2.10 eV) for CTP-3; and (iii) EIS Nyquist plots exhibit the smallest semicircle (lowest Rct) for CTP-3, evidencing the fastest interfacial charge transfer. Collectively, these descriptors rationalize why CTP-3 combines superior photon utilization with efficient charge separation/transport, translating directly into the highest apparent rate constant during MB degradation. Thus, the superior activity of CTP-3 is attributed not only to broadened visible-light absorption but also to stronger interfacial coupling that enables more efficient carrier extraction, lower transfer resistance, and better utilization of the photogenerated charges across the PPy–TiO_2_–CNT junction.

To further assess whether the visible-light activity of the optimized CTP-3 photocatalyst is limited to methylene blue alone, a supplementary photodegradation experiment was additionally carried out using phenol as a second model pollutant under visible light. The test was performed at an initial phenol concentration of 20 mg L^–1^, pH 8.7, and a catalyst dosage of 0.628 mg L^–1^. As shown in Figure S6­(a), the phenol concentration changed only slightly during the dark stage, indicating negligible adsorption-driven removal, whereas a clear and continuous decrease in *C*_t_/*C*_0_was observed after light irradiation. The corresponding pseudo-first-order kinetic plot in Figure S6­(b) showed good linearity, giving an apparent rate constant of 0.6316 ± 0.0356 h^–1^ (≈0.0105 ± 0.0006 min^–1^) with *R* ^2^ = 0.9632. Although this supplementary phenol test was not intended as a full optimization study, it nevertheless provides useful supporting evidence that the optimized CTP-3 photocatalyst is not restricted to a single cationic dye system and retains visible-light activity toward a structurally different, colorless aromatic pollutant.

The superior performance of CTP-3 is therefore attributed to its optimized PPy–TiO_2_–CNT composition, which (i) strengthens visible-light utilization via PPy sensitization and broadened absorption, (ii) promotes charge-carrier separation and suppresses recombination (PL quenching), and (iii) accelerates interfacial charge transfer through a conductive CNT network (low Rct). In addition, literature benchmarking against representative binary PPy–TiO_2_ and PPy–CNT systems indicates that CTP-3 is not only the best performer within the present catalyst series but also a competitive PPy-containing photocatalyst relative to previously reported binary systems. Based on these results, CTP-3 was selected as the optimal catalyst for all subsequent structural, optical, electrochemical, and mechanistic investigations in this study, owing to its outstanding photocatalytic kinetics and consistent multitechnique evidence of enhanced photophysical/electrochemical behavior.

### 3.9 Investigation of Photocatalytic Performance

#### 3.9.1 Effect of Catalyst Dosage

The influence of catalyst loading (0.1, 0.4, 0.7, and 1.0 mg mL^–1^) on MB degradation is shown in Figure 7a. Increasing the photocatalyst dosage markedly enhanced the removal efficiency. At 1.0 mg mL^–1^, nearly complete degradation was achieved within 1 h of visible light exposure, whereas at 0.1 mg mL^–1^, only partial removal was recorded. This improvement can be attributed to the increased number of available active sites and the higher light-harvesting capacity at higher loadings.^65^ However, the marginal gain between 0.7 mg mL^–1^ and 1.0 mg mL^–1^ suggests a saturation effect, where light scattering and particle agglomeration may limit further improvement.^66^

**Figure 7:**
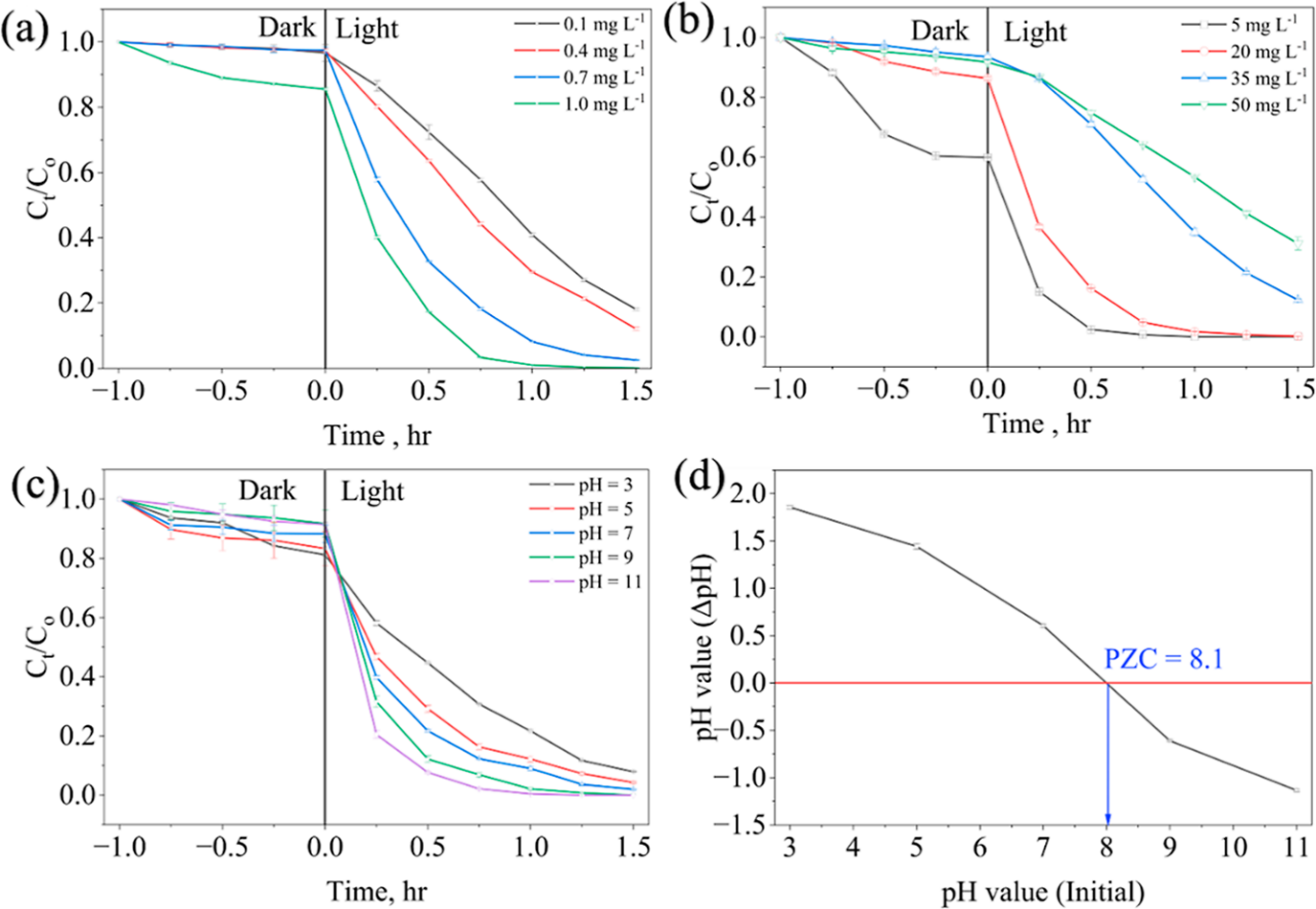
Effect of operational parameters on the photocatalytic degradation of MB by PPy–TiO_2_–CNT nanocomposite under visible light irradiation: (a) effect of catalyst dosage (0.1–1.0 mg mL^–1^), (b) effect of initial MB concentration (5–50 mg L^–1^), (c) effect of solution pH (3–11), and (d) determination of the point of zero charge (PZC = 8.1) of the photocatalyst.

#### 3.9.2 Effect of Initial MB Concentration

The role of initial MB concentration (5, 20, 35, and 50 mg L^–1^) is illustrated in Figure 7b. At the lowest concentration (5 mg L^–1^), a complete degradation was achieved in less than 1 h, while higher concentrations slowed the degradation rate. This is likely due to the increased competition among dye molecules for active sites and the higher light absorption by the dye itself, reducing the photons reaching the catalyst surface.^67^ At 50 mg L^–1^, ∼85% removal was recorded after 1.5 h, indicating that while the photocatalyst remains effective at high concentrations, the rate constant decreases with concentration due to photon attenuation and surface saturation.^68^

#### 3.9.3 Effect of pH

The degradation performance at different pH values (3, 5, 7, 9, and 11) is presented in Figure 7c. The photocatalyst showed high activity across the pH range, with optimal removal observed under alkaline conditions (pH 9 and 11). To explain this trend, the point of zero charge (PZC) of the PPy–TiO_2_–CNT nanocomposite was determined using the pH drift method (Figure 7d). The PZC was found to be 8.1 ± 0.1, indicating that at pH > 8.1, the catalyst surface acquires a net negative charge. This electrostatically attracts the positively charged MB molecules, enhancing adsorption and subsequent photocatalytic degradation.^10^ This explains the superior performance observed at pH 9 and 11. Conversely, at pH < 8.1, the surface is positively charged, leading to electrostatic repulsion with cationic MB and a corresponding reduction in degradation efficiency.^9^

### 3.10 Scavenger Photodegradation

Radical-trapping tests were conducted to elucidate the dominant reactive species and the operative charge-carrier pathway governing MB removal over CTP-3. As shown in Figure 8a,b, in the absence of scavengers, MB degradation reaches ∼99.8%, confirming the high intrinsic photocatalytic activity of the selected composite under visible light irradiation. Upon adding isopropanol (IPA; ^•^OH scavenger), the degradation efficiency is drastically suppressed to ∼15%, indicating that ^•^OH radicals are the dominant oxidizing species under the present conditions.

**Figure 8:**
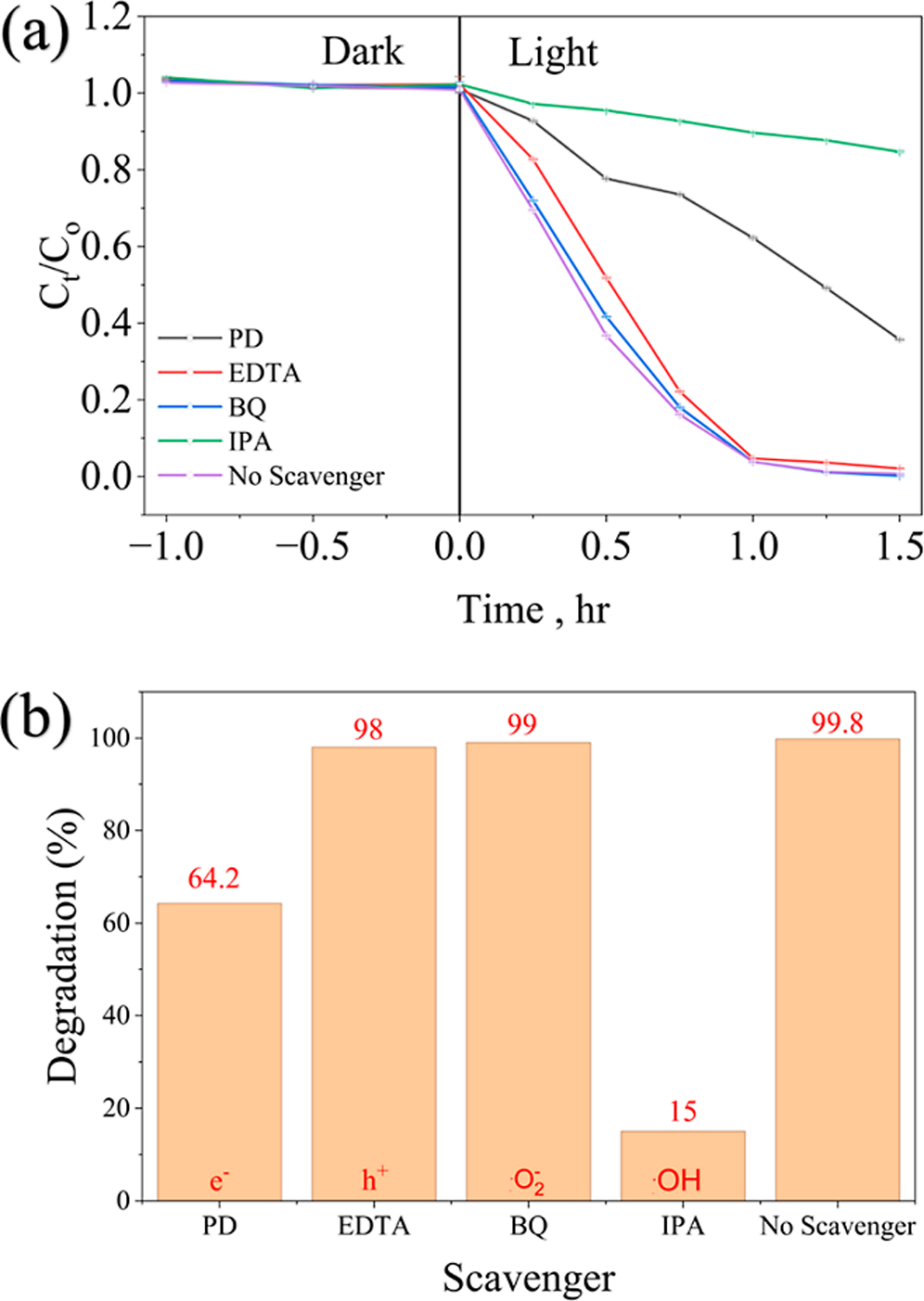
Scavenger effects on MB photodegradation over CTP-3 (visible light). (a) *C*_t_/*C*_0_ vs time with the different scavengers: PD (e^–^), EDTA (h^+^), BQ (^•^O_2_^–^), IPA (·OH), and no scavenger and (b) Degradation efficiency with the different scavengers: PD (e^–^), EDTA (h^+^), BQ (·O_2_^–^), IPA (·OH), and no scavenger.

The addition of potassium dichromate (PD; electron scavenger) decreases MB removal to ∼64.2%, indicating that photogenerated electrons play an important role in the reduction–side reactions that contribute to downstream ROS generation. This observation is consistent with strong electron participation in the degradation process, rather than with a mechanism dominated solely by direct hole oxidation.^69^ In contrast, EDTA (hole scavenger) and benzoquinone (BQ; ^•^O_2_^–^scavenger) maintain high degradation efficiencies (∼98–99%), suggesting that direct hole oxidation is not the rate-controlling pathway and that freely diffusing superoxide is unlikely to be the dominant terminal oxidant in bulk solution. However, the negligible effect of BQ should be interpreted cautiously.

The present scavenger data do not provide direct proof that ^•^O_2_^–^ is a confirmed intermediate under the present conditions. In addition to any possible short-lived oxygen-reduction species, the weak BQ response may also reflect limited diffusion or penetration of BQ into the catalyst layer/interfacial region and possible competition between BQ and MB for adsorption on active sites.^70^ Therefore, the scavenger results support the roles of electrons and ^•^OH, but do not by themselves establish a specific superoxide-mediated route. Taken together, the scavenger responses suggest that an oxygen-involved electron-assisted pathway is plausible, in which electron transfer to dissolved O_2_ may contribute to subsequent ROS conversion steps leading ultimately to ^•^OH, which governs the final oxidation steps responsible for dye decolorization and breakdown. Accordingly, eq 5 is presented here as a plausible ROS-generation sequence rather than as a directly proven elementary mechanism

5 $$O_{2}{+e}^{-}{{{\to \cdot O}_{2}}_{2}}^{-}{\to HO}_{2}{\cdot \to H}_{2}O_{2}\to \left(e^{-}/h\nu \right)\to \cdot OH$$

Thus, the scavenger data most strongly support two conclusions: (i) ^•^OH is the dominant oxidizing species and (ii) photogenerated electrons play a significant role in enabling the oxidative pathway.

### 3.11 Photocatalytic Degradation Mechanism

Under visible light, the PPy-CNT-TiO_2_ composite generates electron–hole pairs in both phases: electrons are promoted from the HOMO to the LUMO in polypyrrole (PPy) and from the TiO_2_ valence band (VB) to its conduction band (CB), establishing e^–^/h^+^ populations available for surface reactions. The CNT network serves as a solid-state electron mediator that electronically couples PPy and TiO_2_, accelerates interfacial carrier exchange, and suppresses bulk recombination, thereby preserving the most reactive carriers for redox steps at the interface.^71–73^ Accordingly, Scheme 2 is used to visualize (i) the relative energy positions of PPy (HOMO/LUMO) and TiO_2_ (VB/CB) on the NHE scale and (ii) the dominant charge-migration directions and interfacial redox steps responsible for ROS formation and MB oxidation.

**Scheme 2:**
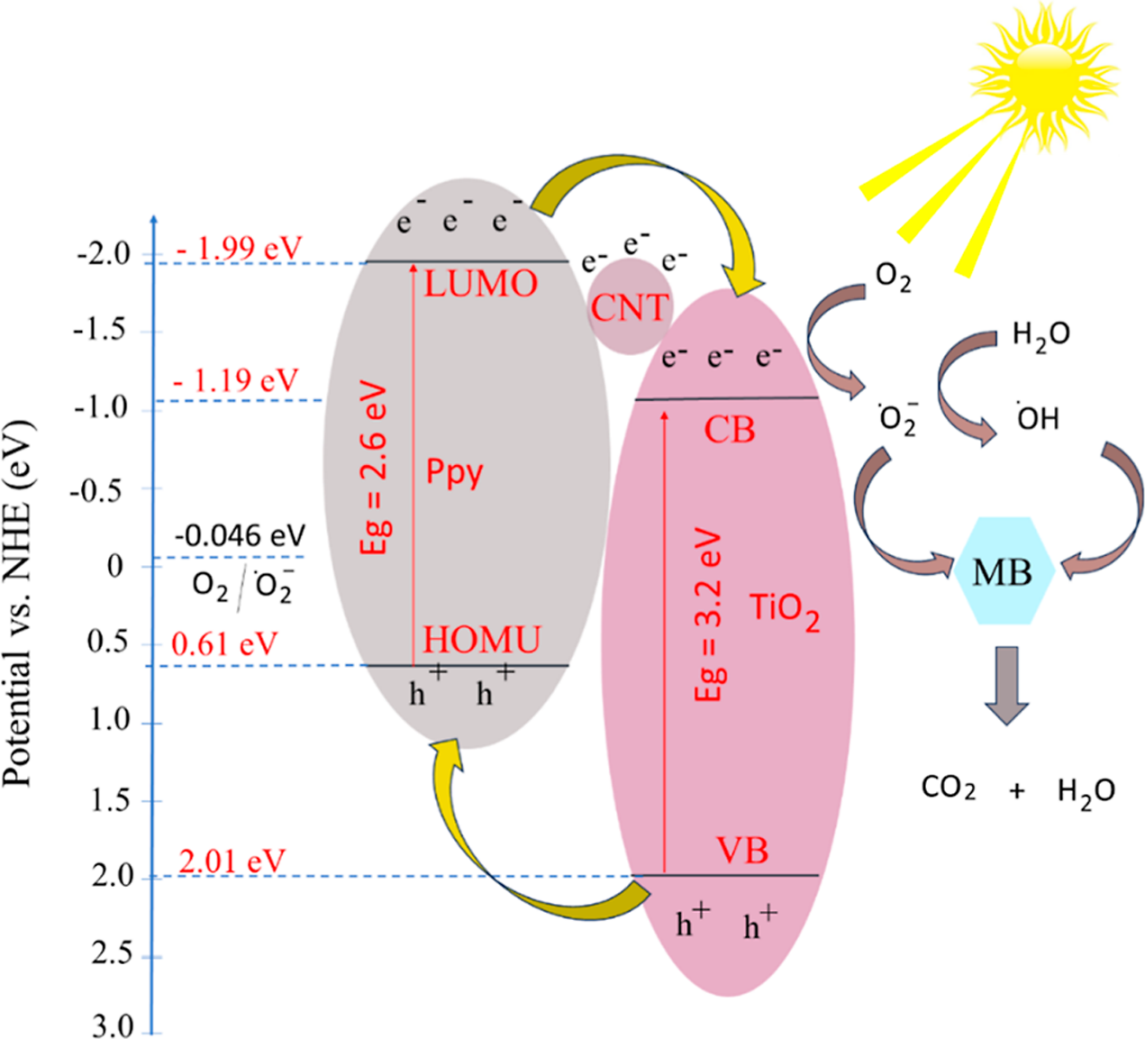
Visible-Light Band Alignment and Charge Transfer in PPy–TiO_2_–CNT

As shown in Figure S7a–c, the valence-band (VB) onsets were taken from the VB-XPS spectra by linear extrapolation of the leading edge to the baseline, yielding E_VB,XPS_ values of 1.63 eV (CTP-3), 2.25 eV (TiO_2_), and 0.85 eV (PPy). These values were converted to the NHE scale using eq 6 ^74,75^

6 EVB,NHE=ϕ+EVB,XPS−4.44

where φ is the analyzer work function (taken here as ≈4.2 eV). Based on this conversion, the VB position of CTP-3 is estimated to be ≈ + 1.39 V vs NHE. Combining this experimentally anchored VB value with the optical band gap (Eg = 2.10 eV) gives an estimated CB position of ≈ −0.71 V vs NHE. Here, the VB position of CTP-3 is experimentally anchored by VB-XPS, whereas the CB position is a derived estimate based on the measured optical band gap; accordingly, these values are used for qualitative band alignment and redox feasibility discussion rather than as directly measured absolute band-edge positions. The band positions obtained and derived from the present work are summarized in Table 3.

**Table 3:** Band Positions of PPy and CTP-3 Obtained or Derived From the Present Work

sample	*E*_VB,XPS_ (eV)	φ (eV)	*E*_VB,NHE_ (V)	Eg (eV)	*E*_CB,NHE_ /LUMO (eV)
PPy	0.85	4.2	+0.61	2.60	–1.99
CTP-3	1.63	4.2	+1.39	2.10	–0.71

These experimentally anchored energy levels indicate that photoexcited electrons in PPy possess sufficient reducing power to participate in oxygen activation, while the CTP-3 band structure supports efficient interfacial charge separation and visible light-driven ROS formation.

Under visible irradiation, PPy and TiO_2_ generate carriers (eqs 7 and 8). As illustrated in Scheme 2, PPy acts as the primary visible-light sensitizer (HOMO → LUMO), while CNT provides a conductive percolation pathway that collects and transports photogenerated electrons, reducing recombination and facilitating electron delivery to interfacial acceptors. Importantly, TiO_2_ is expected to contribute mainly through interfacial charge separation/electron acceptance and through the higher-energy fraction of the xenon-lamp spectrum, rather than through strong intrinsic visible-light absorption. Thus, the electron-transfer arrows in Scheme 2 represent a preferred electron-flow route from photoexcited PPy to the CNT network and then to dissolved O_2_ at the catalyst/solution interface.

When considered together, the scavenger results and the band energetics indicate that MB degradation proceeds mainly through an electron-assisted oxygen-reduction pathway, while ^•^OH acts as the dominant oxidizing species. This interpretation is consistent with the strong inhibition observed with PD and IPA, which shows that electron participation and ^•^OH formation is both critical to the reaction, as reflected in eqs 9–12. In contrast, the weak suppression observed by EDTA suggests that direct hole oxidation is not the rate-controlling pathway. More importantly, this conclusion is also supported thermodynamically: the TiO_2_ VB position (≈+ 2.01 V vs NHE) is less positive than the OH^–^/·OH potential (+2.68 V vs NHE),^76^ indicating that direct hole-driven generation of ^•^OH is not energetically favorable under the present conditions. Thus, the weak effect of hole scavenging is consistent with the band-alignment argument rather than with a hole-dominated oxidation route. Because the PPy LUMO (≈ −1.99 V vs NHE) is more negative than E°(O_2_/·O_2_^–^) (≈ −0.046 V),^66^ electrons preferentially accumulate on PPy and reduce dissolved O_2_ to ^•^O_2_^–^, as shown in eq 9, which can proceed via ^•^HO_2_ and H_2_O_2_ to ^•^OH through eqs 10–12. In contrast, the TiO_2_ VB (≈+ 2.01 V vs NHE) is thermodynamically insufficient to oxidize OH^–^ to ^•^OH (+2.68 V vs NHE),^77^ providing a mechanistic rationale for the weak suppression observed with hole scavenging and supporting an electron-driven ROS cascade.

The proposed reaction chain is detailed as follows:

Photoexcitation

7 $$PPy+h\nu \to e^{-}\left(LUMO\right)+h^{+}\left(HOMO\right)$$

8 $${TiO}_{2}+h\nu \to e^{-}\left(CB\right)+h^{+}\left(VB\right)$$

Oxygen Reduction Chain (Electron Pathway)

9 $$O_{2}{+e}^{-}{{\left(LUMO\;of\;Ppy\right)\to \cdot O}_{2}}^{-}$$

10 $${{{\cdot O}_{2}}_{2}}^{-}{+H}^{+}⇌{\cdot HO}_{2}$$

11 $${2\cdot HO}_{2}{\to H}_{2}O_{2}{+O}_{2}$$

12 $$H_{2}O_{2}{+e}^{-}{\to \cdot OH+OH}^{-}$$

Target Oxidation

13 $$ROS\left(\cdot {O_{2}}^{-},\cdot OH\right)+MB\to Intermediates\to CO_{2}{+H}_{2}O$$

Therefore, the role of CNT is to mediate rapid electron transport across the PPy/TiO_2_ interfaces and toward dissolved O_2_, increasing the probability of O_2_ reduction (Equation 9) relative to recombination. Conversely, the VB position of TiO_2_ relative to the OH^–^/^•^OH potential constrains direct hole-driven ^•^OH formation, explaining why holes contribute minimally to the overall MB degradation under the present band alignment. In summary, eqs 7–12 describe an electron-mediated ROS-generation sequence initiated by photoexcited PPy and facilitated by CNT-assisted charge transport, whereas eq 13 represents the subsequent oxidation of MB by the generated ROS. The combined energetic analysis and scavenger evidence therefore support an electron-driven degradation pathway, while direct hole-derived ^•^OH formation is disfavored.

### 3.12 Proposed Reaction Pathway

Liquid chromatography–mass spectrometry (LC–MS) was used to identify MB degradation intermediates formed over CTP-3 under visible light. Aliquots collected at 0, 15, 30, 45, and 60 min are denoted MB0–MB4, respectively. Figure S8 presents the ESI^+^ LC–MS full-scan spectra (*m*/*z* 50–600) for the time-resolved aliquots MB0–MB4 (0, 15, 30, 45, 60 min), showing the decay of the parent MB ion (*m*/*z* ≈ 284) and the sequential appearance/decay of fragments at *m*/*z* ≈ 270, 256, 242, 228 followed by 201, 184, 110, 94, 68, and 55 consistent with the N-demethylation → ring-opening → deep-oxidation sequence summarized in Scheme 3.

**Scheme 3:**
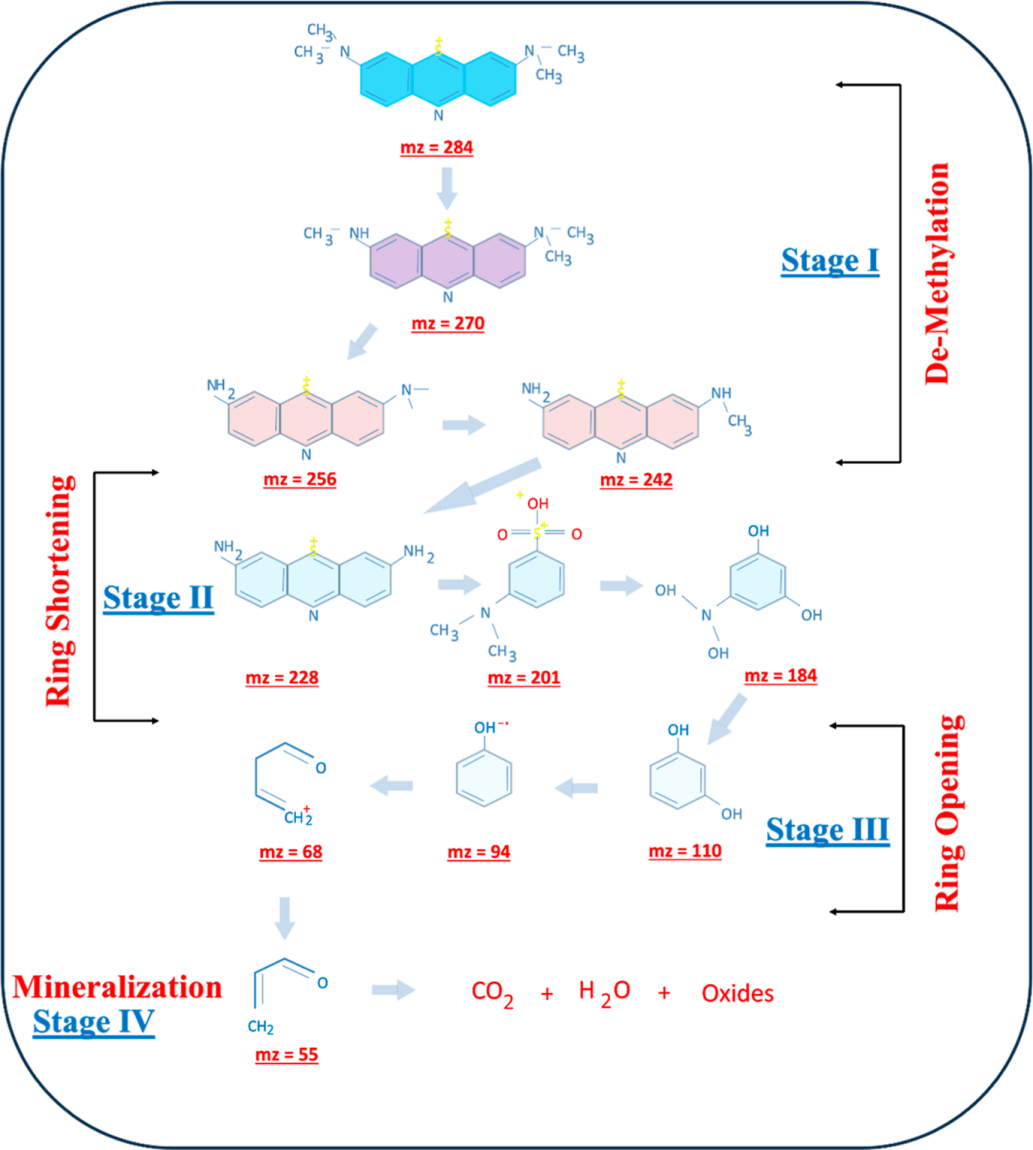
Proposed LC–MS-Supported Photodegradation Pathway of Methylene Blue (MB) Under Visible Light

The detailed identification of these intermediates, including their proposed formulas and corresponding literature assignments, is summarized in Table S4. Scheme 3 summarizes a four-stage pathway derived from these data. Stage I (N-demethylation) is visualized in Scheme 3 at the top of the pathway (demethylation), where the parent MB ion (*m*/*z* ≈ 284) yields sequential demethylated products (*m*/*z* ≈ 270, 256, 242, 228).

In the initial stage (I), the parent ion at *m*/*z* ≈ 284 diminishes while new ions appear at *m*/*z* ≈ 270, 256, 242, and 228, consistent with sequential N-demethylation prior to ring disruption.^78^ Stage II (ring opening/cleavage) is visualized in Scheme 3 in the middle-right portion (Ring Opening), corresponding to the emergence of lower *m*/*z* intermediates (201 and 184) observed in Figure S8 as irradiation proceeds (MB2–MB3). With continued irradiation, these intermediates undergo further oxidation and structural contraction of the conjugated framework. indicating aromatic ring opening/cleavage.^79^ Stage III (deep oxidation/ring shortening) is visualized in Scheme 3, along the left/middle branch (Ring Shortening), where smaller aromatic/phenolic fragments form (*m*/*z* ≈ 110, 94, and 68), consistent with the fragment pattern in the later spectra (MB3–MB4). Stage III reflects deep oxidation, yielding small phenolic/phenyl and aliphatic fragments (≈110, 94, and 68). Stage IV (mineralization) is visualized at the bottom of Scheme 3 (Mineralization), where the pathway converges to CO_2_ and H_2_O; experimentally, this stage is supported by the progressive attenuation of organic fragment signals toward baseline in Figure S8 at longer irradiation times. Stage IV (mineralization) is evidenced by the progressive loss of all fragment signals toward baseline, consistent with conversion of residual organics to CO_2_ and H_2_O under the present conditions.

### 3.13 Recycling Performance of Photocatalyst

The stability and reusability of a photocatalyst are critical metrics for its practical application in sustainable and cost-effective water treatment. To evaluate these properties, the performance of the CTP-3 photocatalyst was assessed over five consecutive cycles of MB degradation. As shown in Figure 9a, the *C*_t_/*C*_0_ profiles remain closely overlapping across cycles, and, as shown in Figure 9b, the photocatalyst demonstrated exceptional initial activity, achieving 99.4% degradation in the first cycle. This high performance was largely maintained through subsequent uses, with the efficiency remaining at 92.3% after the fifth cycle. The minor 7.1% decrease in efficiency over the five runs may be attributed to the partial fouling of the catalyst’s surface by residual dye molecules or intermediate products, which can block active sites and slightly impede light absorption.

**Figure 9:**
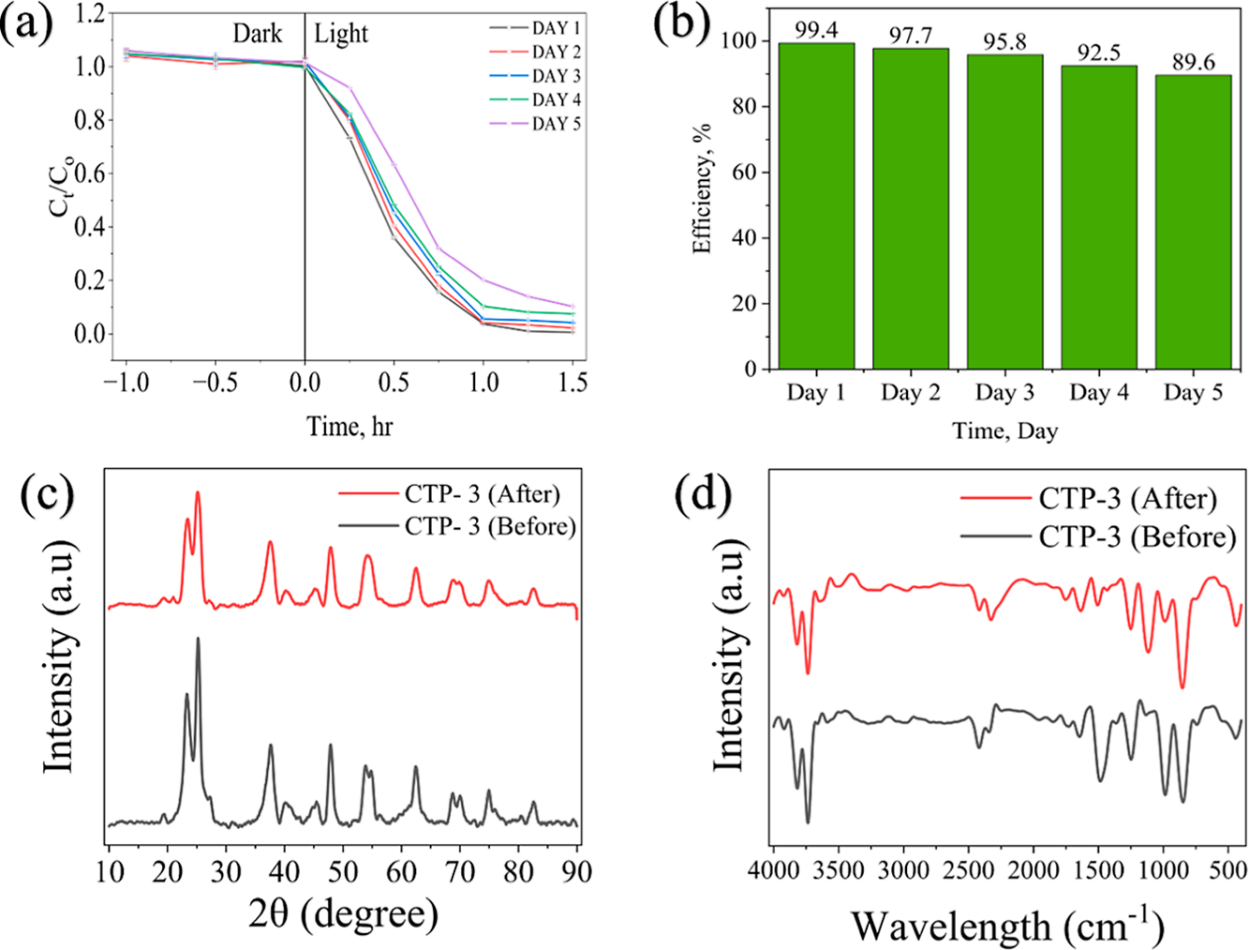
Reusability and structural integrity of CTP-3 during MB photodegradation (a) C_t_/C_0_ decay curves; (b) degradation efficiency % (Days 1–5); (c) XRD before/after showing no new phases; and (d) FTIR before/after with preserved functional groups.

To verify that this operational stability was rooted in the material’s structural durability, the catalyst was analyzed postcycling. Postcycling analyses confirm material robustness: Figure 9c (XRD) shows unchanged anatase reflections with no new phases or broadening, while Figure 9d (FTIR) preserves the characteristic PPy and TiO_2_ bands without degradation signatures. Together, these findings confirm that the photocatalyst possesses robust physicochemical integrity, allowing for effective reuse with minimal loss in activity and making it a promising candidate for large-scale wastewater treatment applications.

## 4 Conclusion

This study addressed key visible-light photocatalysis bottlenecks, namely band-structure limitations, weak interfacial coupling, and sluggish charge transport that accelerate electron–hole recombination through two main objectives: (i) constructing a continuously coupled PPy–TiO_2_–CNT heterojunction with intimate polymer/oxide/carbon contact to promote charge separation and carrier transport, and (ii) systematically optimizing operating conditions (catalyst dosage, initial MB concentration, and pH) to define the high-performance window for MB removal. The outcomes confirm that the PPy–TiO_2_–CNT heterojunction design effectively mitigates recombination and charge-transport limitations. The CTP-3 composition delivered the fastest kinetics for methylene blue (MB) degradation (*k* = 0.053 min^–1^ and 99.4% removal) and retained 92.3% activity after five cycles, evidencing durability. The strongest PL quenching together with the lowest EIS charge-transfer resistance (R_ct) among the prepared compositions indicates improved interfacial charge separation and faster carrier transport, consistent with suppressed recombination enabled by the continuously coupled junction. DRS/Tauc further indicates an apparent optical gap of ≈2.10 eV, supporting efficient visible-light harvesting. In addition, the visible-light phenol degradation test indicates that the optimized CTP-3 photocatalyst is not limited to methylene blue alone, thereby supporting broader photocatalytic applicability within the scope of the present study.

VB-XPS-anchored valence-band positions, together with optical-gap-derived band-edge estimates, support a qualitative redox-feasibility picture for the optimized heterojunction rather than exact quantitative assignment of all charge-transfer steps. Scavenger experiments identify ^•^OH as the dominant oxidizing species and indicate significant electron participation in the degradation process; however, the exact upstream oxygen-reduction intermediates cannot be conclusively assigned from the present scavenger data alone. Accordingly, the mechanistic interpretation is best viewed as evidence-supported but not fully definitive, and direct ROS detection techniques such as EPR/ESR would be valuable to further verify the proposed oxygen-involved pathway.

LC–MS mapping corroborates a sequential N-demethylation → aromatic oxidation → ring opening → mineralization pathway, thereby linking the optimized interfacial charge-handling characteristics of CTP-3 to the observed degradation sequence.

In summary, mechanism-guided construction of a continuously coupled PPy–TiO_2_–CNT heterojunction, combined with operating-window optimization, provides a durable and efficient visible-light photocatalyst and a reproducible route for materials–process codesign in water remediation applications. At the same time, the present study has several limitations that should be acknowledged. First, no surface area or porosity analysis (e.g., BET) was performed, which limits more rigorous assessment of how accessibility and textural properties contribute to the observed activity. Second, although the five-cycle test indicates short-term reusability, the stability evaluation remains limited and does not yet address long-term durability, structural evolution, or possible material degradation during extended photocatalytic operation. Third, because the mechanistic analysis relies mainly on scavenger tests, band alignment, and LC–MS pathway evidence, direct ROS verification was beyond the scope of the present work. These aspects should be examined in future work to strengthen structure–property–stability correlations and further validate the practical applicability of the PPy–TiO_2_–CNT system.

## Supplementary Material



## Notes

Complementary physicochemical, electronic, and pathway evidence that underpins the proposed PPy–TiO_2_–CNT heterojunction architecture and its visible-light photocatalytic function; fitted anatase TiO_2_ (101) peak positions, fwhm values, and corresponding Scherrer crystallite sizes for the PPy–TiO_2_–CNT nanocomposites (CTP-1 to CTP-4), confirming nanoscale crystalline domain formation within the hybrid framework; XPS confirming the concurrent presence of C/N (PPy/CNT) and Ti/O (TiO_2_) in CTP-3 and verifying the expected chemical-state signatures from the survey and high-resolution C 1s, N 1s, Ti 2p, and O 1s regions; fitted binding energies, fwhm values, relative peak areas, and assignments obtained from Shirley-background fitting of the high-resolution XPS spectra of CTP-3, providing enhanced insight into lattice oxygen contributions and PPy-derived nitrogen environments; TGA/DTG summarizing the composition-dependent thermal evolution of the CTP series (CTP-1-CTP-6 ; CTP-6 = PPy), providing additional confirmation of polymer incorporation and relative component contributions through characteristic mass-loss profiles; TEM documenting the nanoscale morphology and component integration across the series, supporting the formation of intimately coupled hybrid domains rather than simple physical mixtures; UV–Vis DRS/Tauc reporting band gap determinations for CTP-1-CTP-6 and corroborating the extended visible-light absorption of the optimized formulation; EIS Nyquist comparing interfacial charge-transfer resistance across the CTP series, supplying electrochemical evidence for composition-dependent carrier-transport behavior; phenol photodegradation over the optimized CTP-3 photocatalyst under dark adsorption and subsequent visible-light irradiation conditions, together with the corresponding pseudo-first-order kinetic plot, offering an independent kinetic validation of photocatalytic performance beyond dye degradation; present ternary PPy–TiO_2_–CNT system against representative binary PPy–TiO_2_ and PPy–CNT photocatalysts reported for organic pollutant degradation, highlighting the improved degradation kinetics achieved in this work; time-resolved LC–MS tracking the evolution of the parent MB signal and transient intermediates during irradiation (0–60 min), supporting the proposed sequential transformation pathway under visible-light conditions; tentative LC–MS assignments for the principal intermediates detected during methylene blue (MB) photodegradation over CTP-3 under visible light, based on the present full-scan ESI^+^ data and literature-reported degradation pathways, with the experimentally observed early-stage N-demethylation sequence (*m*/*z* 284 → 270 → 256 → 243 → 228); and corresponding time-resolved LC–MS spectra (0–60 min), tracking the evolution of the parent MB signal and transient intermediates to support the proposed sequential transformation pathway involving demethylation, ring opening, deep oxidation, and eventual mineralization under visible-light conditions (PDF)

## Abbreviations

AOPs: advanced oxidation processes
CB: conduction band
CCD: central composite design
COD: chemical oxygen demand
Co: initial concentration (after dark equilibration)
Ct: concentration at time t
CNT: carbon nanotube(s)
CTP-x (CTP-1 to CTP-6): CNT–PPy–TiO_2_ photocatalyst series labels
DI: deionized
DRS: diffuse reflectance spectroscopy
DTG: derivative thermogravimetry
EDX/EDS: energy-dispersive X-ray spectroscopy
Eg: optical band gap; EIS, electrochemical impedance spectroscopy
ESI^+^: electrospray ionization (positive mode)
FEG: field-emission gun
FTO: fluorine-doped tin oxide
FTIR: Fourier transform infrared spectroscopy
HOMO: highest occupied molecular orbital
HRTEM: high-resolution transmission electron microscopy
IPA: isopropanol
ID/IG: Raman D-band to G-band intensity ratio
JCPDS: Joint Committee on Powder Diffraction Standards
KNO_3_: potassium nitrate
KOH: potassium hydroxide
k (kapp): apparent pseudo-first-order rate constant
LC–MS: liquid chromatography–mass spectrometry
LUMO: lowest unoccupied molecular orbital
MB: methylene blue
*m*/*z*: mass-to-charge ratio;
NaOH: sodium hydroxide
NHE: normal hydrogen electrode
Olat: lattice oxygen
ORCID: open researcher and contributor ID
PD: potassium dichromate
PL: photoluminescence
PZC: point of zero charge
pH_p_zc: pH at the point of zero charge
PPy: polypyrrole
Py: pyrrole
PTFE: polytetrafluoroethylene;
Rct (Rct): charge-transfer resistance
ROS: reactive oxygen species
*R* ^2^: coefficient of determination
rpm: revolutions per minute
SEM: scanning electron microscopy
TGA: thermogravimetric analysis
TiO_2_: titanium dioxide
UPLC: ultraperformance liquid chromatography
UV: ultraviolet
UV–Vis: ultraviolet–visible
VB: valence band
VB-XPS: valence-band X-ray photoelectron spectroscopy
XPS: X-ray photoelectron spectroscopy
XRD: X-ray diffraction. Symbols used: e^–^, electron
h^+^: hole
•O_2_^–^: superoxide radical anion
HO_2_•: hydroperoxyl radical
H_2_O_2_: hydrogen peroxide
•OH: hydroxyl radical.
