# Supplementary material for: Mechanism-Guided Design of a PPy–TiO2–CNT Nanocomposite for Visible-Light Photodegradation
Source: ACS Omega. 2026 Jun 10;11(24):35827–45. doi: 10.1021/acsomega.6c02369 (PMC13294879; doi:10.1021/acsomega.6c02369)
Supplement: Supplementary file 1 [file ao6c02369_si_001.pdf]

Mechanism-Anchored Design of a PPy–TiO_2–CNT Nanocomposite for Visible-Light Photodegradation

Al-Ali Hussein[1], Soon Huat Tan[1] and Vel Murugan Vadivelu[1]*

[1]School of Chemical Engineering, Universiti Sains Malaysia, Engineering Campus, 14300 Nibong Tebal, Pulau Pinang, Malaysia

Table S1. Fitted anatase TiO_2 (101) peak positions, FWHM values, and corresponding Scherrer crystallite sizes for the PPy–TiO_2–CNT nanocomposites (CTP-1 to CTP-4).

Sample	2θ (°)	FWHM (°)	Crystallite size (nm)
CTP-1	25.3531	0.7909	10.30
CTP-2	25.2341	0.8628	9.44
CTP-3	25.2170	0.7307	11.14
CTP-4	25.0850	0.7657	10.65

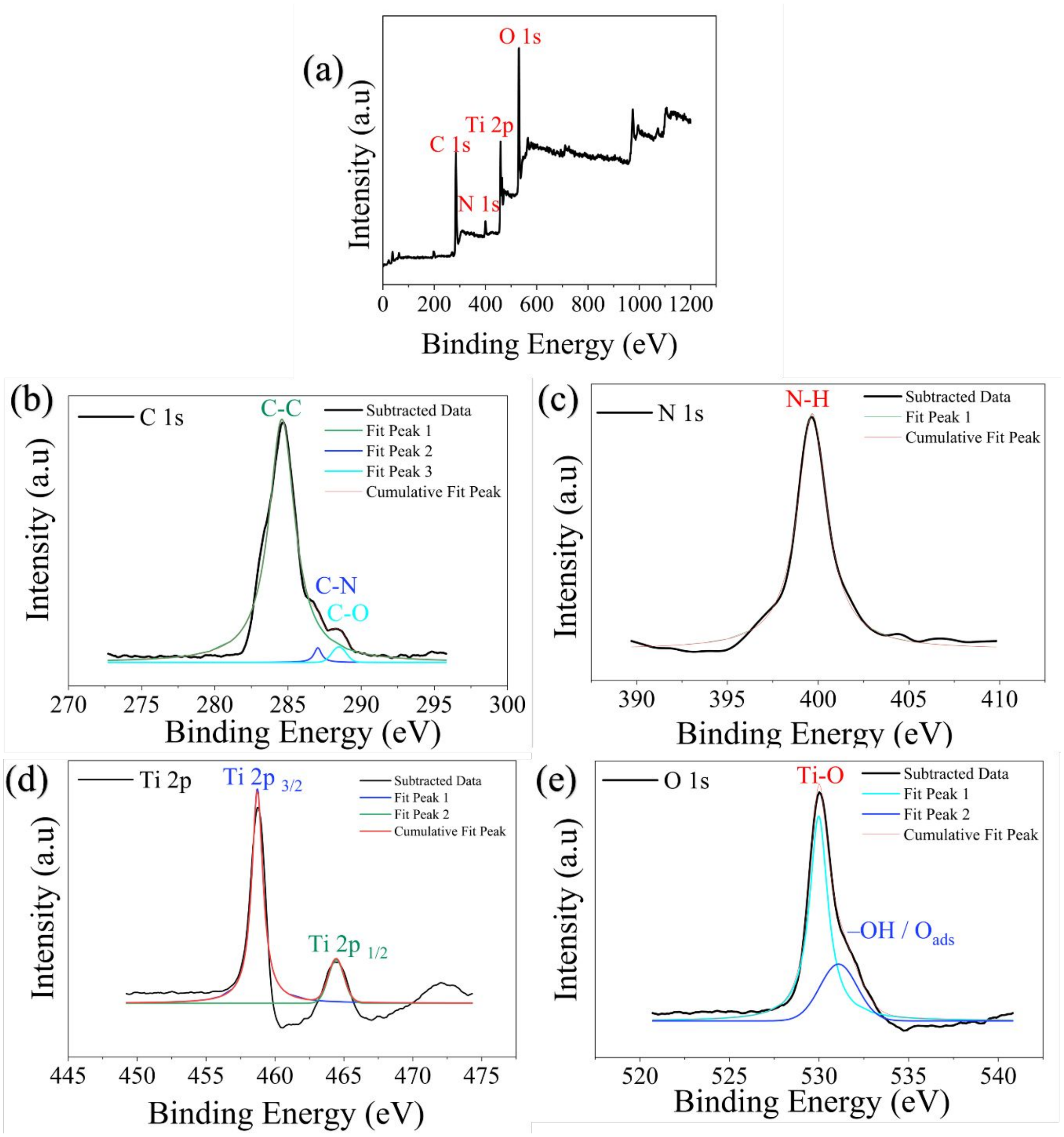

Figure S1. XPS spectra of PPy–TiO_2–CNT (CTP-3): (a) survey; high-resolution (b) C 1s (C–C/C–N/C–O), (c) N 1s (N–H), (d) Ti 2p ($2p_{3/2}$, $2p_{1/2}$), and (e) O 1s (Ti–O).

Table S2. Summary of the fitted binding energies, full width at half maximum (FWHM), relative peak areas, and peak assignments obtained from Shirley-background fitting of the high-resolution XPS spectra of CTP-3.

Region	Component	Binding energy (eV)	Width/FWHM (eV)	Relative area (%)	Assignment
C 1s	Peak 1	284.58	2.16	96.15	C–C/C=C
C 1s	Peak 2	287.04	0.62	1.65	Minor higher-BE carbon species
C 1s	Peak 3	288.48	1.14	2.20	Minor oxidized carbon species
N 1s	Peak 1	399.67	2.01	100.00	PPy-related nitrogen envelope
O 1s	Peak 1	529.98	1.25	73.05	Lattice oxygen (Ti–O)
O 1s	Peak 2	531.09	2.45	26.95	Surface –OH / adsorbed oxygen species
Ti 2p	Ti $2p_{3/2}$	458.72	0.97	84.06	Ti^{4+}
Ti 2p	Ti $2p_{1/2}$	464.44	1.34	15.94	Ti^{4+}

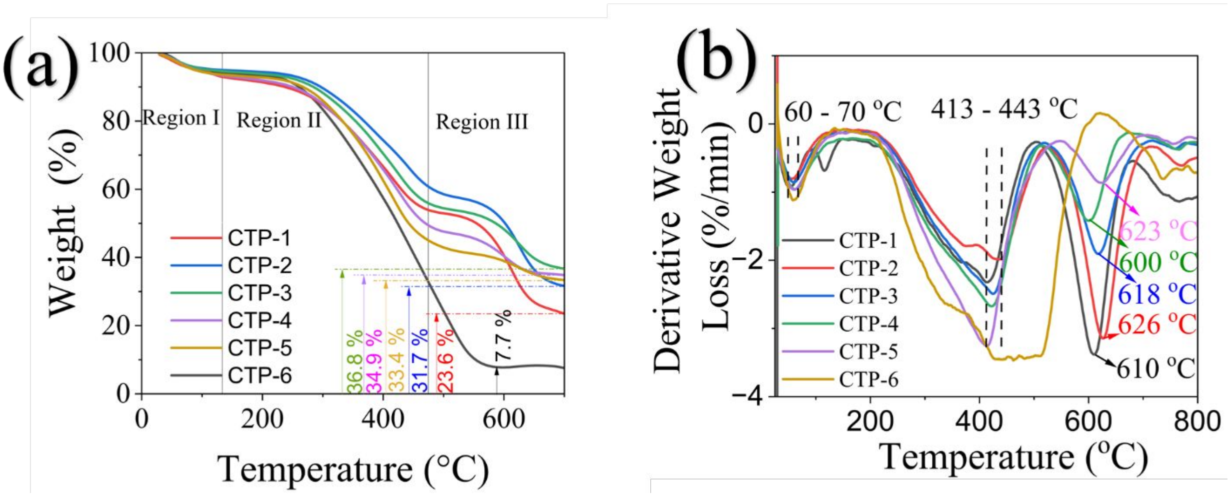

Figure S2. Thermogravimetric (TGA) and derivative profiles of the CTP series (CTP-1–CTP-6; CTP-6 = PPy) measured in air at 10 ˚C min⁻¹. (a) weight losses curves; (b) weight losses derivative curve.

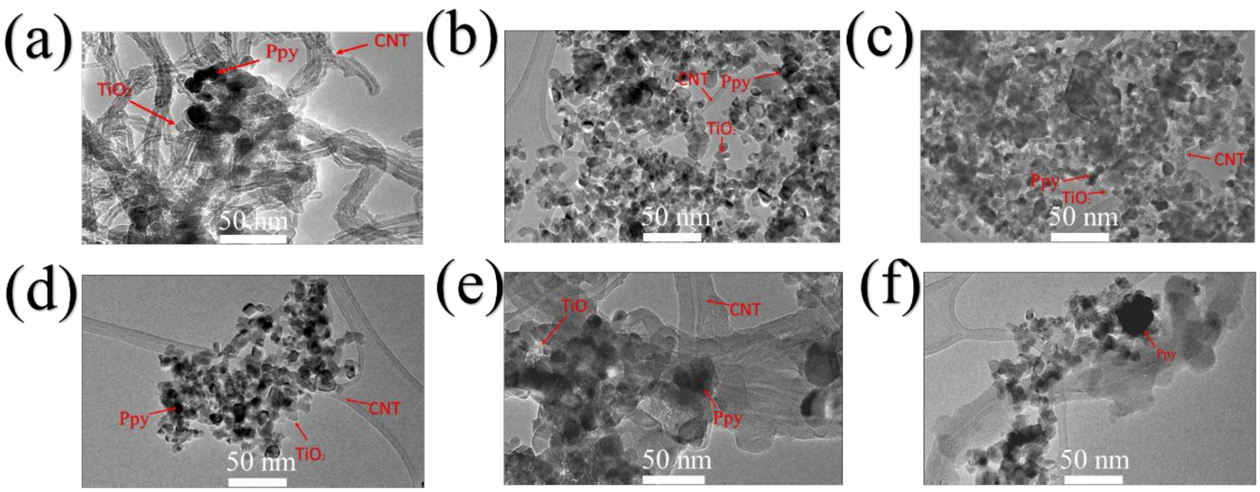

Figure S3. TEM micrographs of PPy–TiO_2–CNT nanocomposites: (a) CTP-1, (b) CTP-2, (c) CTP-3, (d) CTP-4, (e) CTP-5, (f) CTP-6.

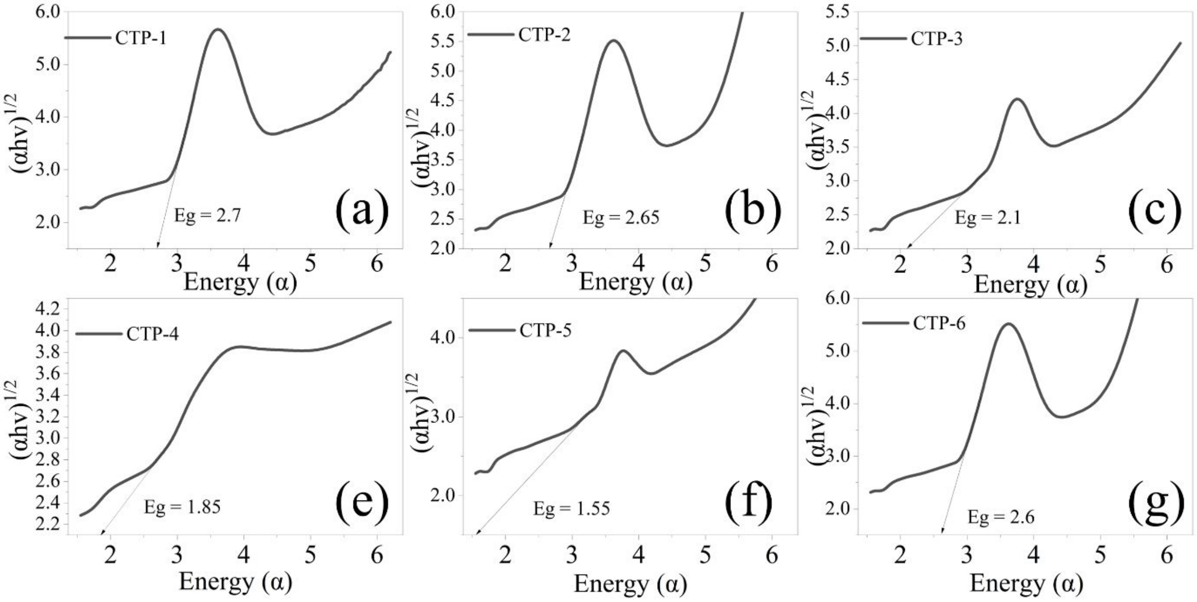

Figure S4. UV–Vis DRS Tauc plots $(\alpha h\nu)^{1/2}$vs $h\nu$ for the CTP series (CTP-1–CTP-6).

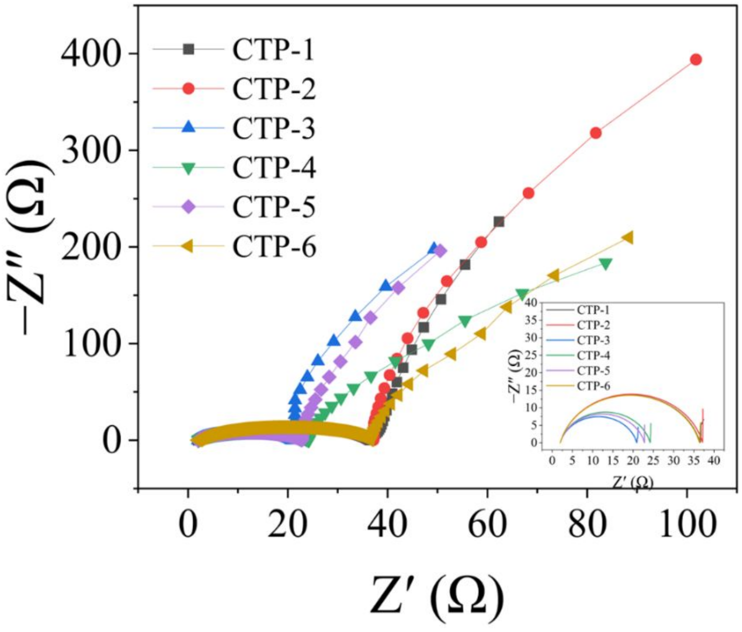

Figure S5. Nyquist Plot Nyquist plots of PPy–TiO_2–CNT nanocomposites (CTP-1 to CTP-6).

Table S3. Comparison of the present PPy–TiO_2–CNT photocatalyst with representative binary PPy–TiO_2 photocatalysts reported for organic pollutant degradation.

System	Nanocomposite	Pollutant	Rate constant	Degradation efficiency	Key operating conditions	Ref.
This work	CTP-3 (PPy–TiO_2–CNT)	Methylene blue	0.0531 min^{-1}	99.4% degradation removal, with 92.3% after 5 cycles	20 mg L^{-1} MB; 0.5 mg mL^{-1} catalyst; 150 min; 500 W Xe lamp	Present work
Binary PPy–TiO_2	PPy/TiO_2	MB	2.19×10^{-2}	95.54% in 120 min	Visible light	[1]

System	Nanocomposite	Pollutant	Rate constant	Degradation efficiency	Key operating conditions	Ref.
Binary PPy–TiO_2	PPy–TiO_2 (0.5–2.0 wt%)	MB	-	93% in 90 min	Solar irradiation; chemical oxidative polymerization	[2]
Binary PPy–TiO_2	Cl-doped PPy@TiO_2	MB	-	58.5% in 30 min; 96% in 120 min	Not clearly reported in attached summary	[3]

System	Nanocomposite	Pollutant	Rate constant	Degradation efficiency	Key operating conditions	Ref.
Binary PPy–TiO_2	TiO_2–PPy	Methyl orange	306.856×10^{-7} M^{-1} s^{-1} *(second-order)*	35% degradation per hour; 43% TOC reduction	One-pot photochemical synthesis	[4]
Binary PPy–TiO_2	Polypyrrole–TiO_2 composite	Diclofenac / 4-chlorophenol	-	>90% diclofenac in 60 min; 40% 4-chlorophenol in 60 min	Simulated solar light	[5]

System	Nanocomposite	Pollutant	Rate constant	Degradation efficiency	Key operating conditions	Ref.
Binary PPy–CNT	PPy–MWCNTs	Methylene blue	-	78.30% in 35 min	Not reported in screened summary	[6]

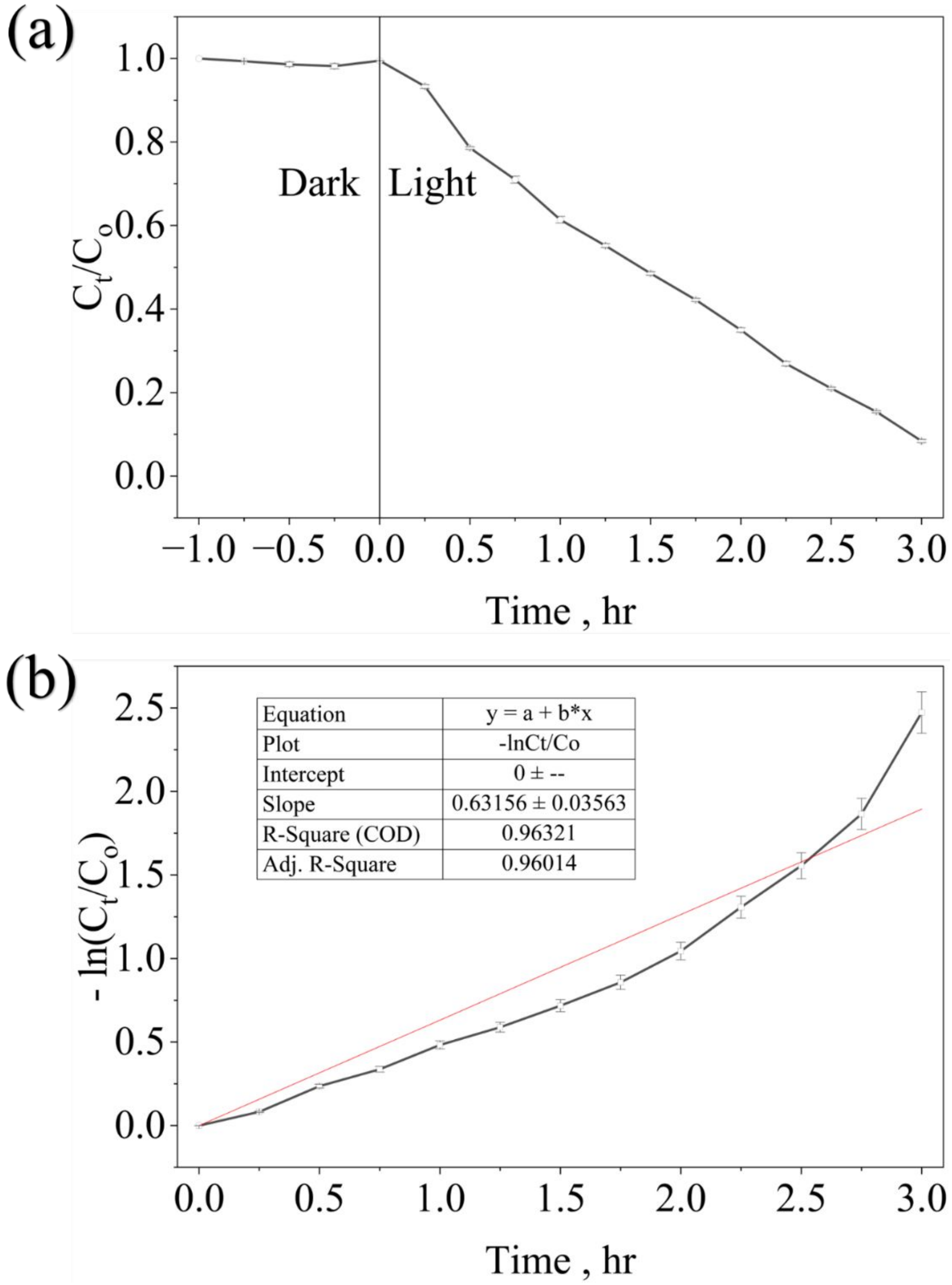

Figure S6. (a) Phenol photodegradation over the CTP-3 photocatalyst under dark adsorption and subsequent light irradiation conditions, expressed as C_t/C_o versus time. (b) Corresponding pseudo-first-order kinetic plot for phenol degradation over CTP-3.

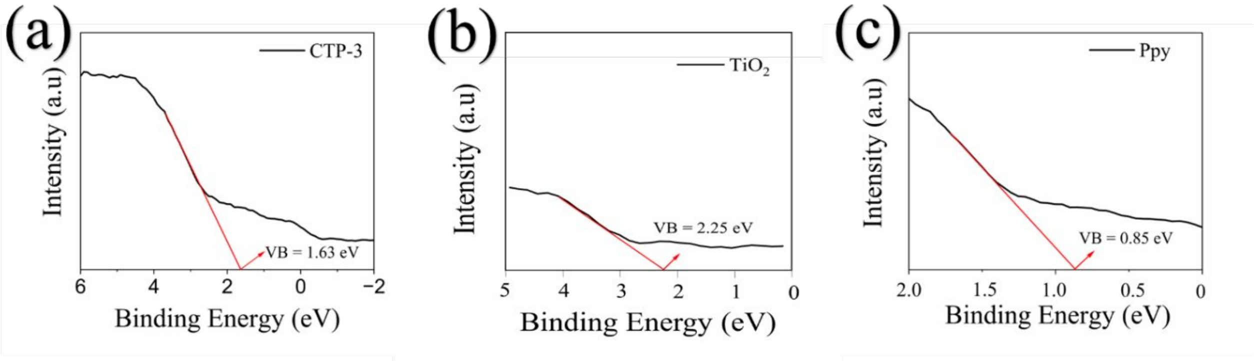

Figure S7. VB-XPS spectra used to locate valence-band onsets by linear extrapolation(a) CTP-3 nanocomposite, (b) TiO_2, and (c) PPy.

Table S4. Tentative LC–MS assignments for the main intermediates detected during MB photodegradation over CTP-3 under visible light.

m/z	Proposed formula assignment /	Assigned intermediate interpretation /	Stage	Ref.
284	$C_{16}H_{18}N_3S^+$	Parent methylene blue cation	Parent dye	[7,8]
270	$C_{15}H_{16}N_3S^+$	Azure B / mono-demethylated	Stage I: N-demethylation	[7–9]

m/z	Proposed formula / assignment	Assigned intermediate / interpretation	Stage	Ref.
		MB-type intermediate		
256	$C_{14}H_{14}N_3S^+$	Azure A / further demethylated MB-type intermediate	Stage I: N-demethylation	[7–9]
242	$C_{13}H_{12}N_3S^+$	Further demethylated MB-type intermediate; closest literature analogue is Azure C at m/z 242	Stage I: N-demethylation	[7,9]
228	Tentative highly demethylated thionine-type intermediate	Late demethylation product; retained as tentative	Stage I: N-demethylation	[8,9]
201	Tentative dimethylaminobenzenesulfonic acid-type fragment	Oxidized aromatic cleavage product	Stage II: Ring opening	[8,9]
184	Tentative oxygenated aromatic amine / diphenylmethane-type fragment	Ring-opened oxidized intermediate	Stage II–III	[9,10]
110	Tentative small oxygenated aromatic fragment	Small aromatic/oxygenated fragment	Stage III: Deep oxidation	[8]

m/z	Proposed formula / assignment	Assigned intermediate / interpretation	Stage	Ref.
		after oxidative cleavage		
94	C_6H_6O (tentative)	Phenol-type / small aromatic fragment	Stage III: Deep oxidation	7
68	Tentative low-mass oxygenated fragment	Deep-oxidation product	Stage III: Deep oxidation	10
55	Tentative low-mass oxygenated fragment	Final small organic fragment before mineralization	Stage IV: Mineralization	10

Note: Assignments are proposed from the present full-scan ESI^+ LC–MS data, the time-dependent evolution of peaks in Figure S8, and comparison with previously reported MB degradation pathways. The experimentally observed early-stage demethylation sequence is m/z 284 → 270 → 256 → 243 → 228.

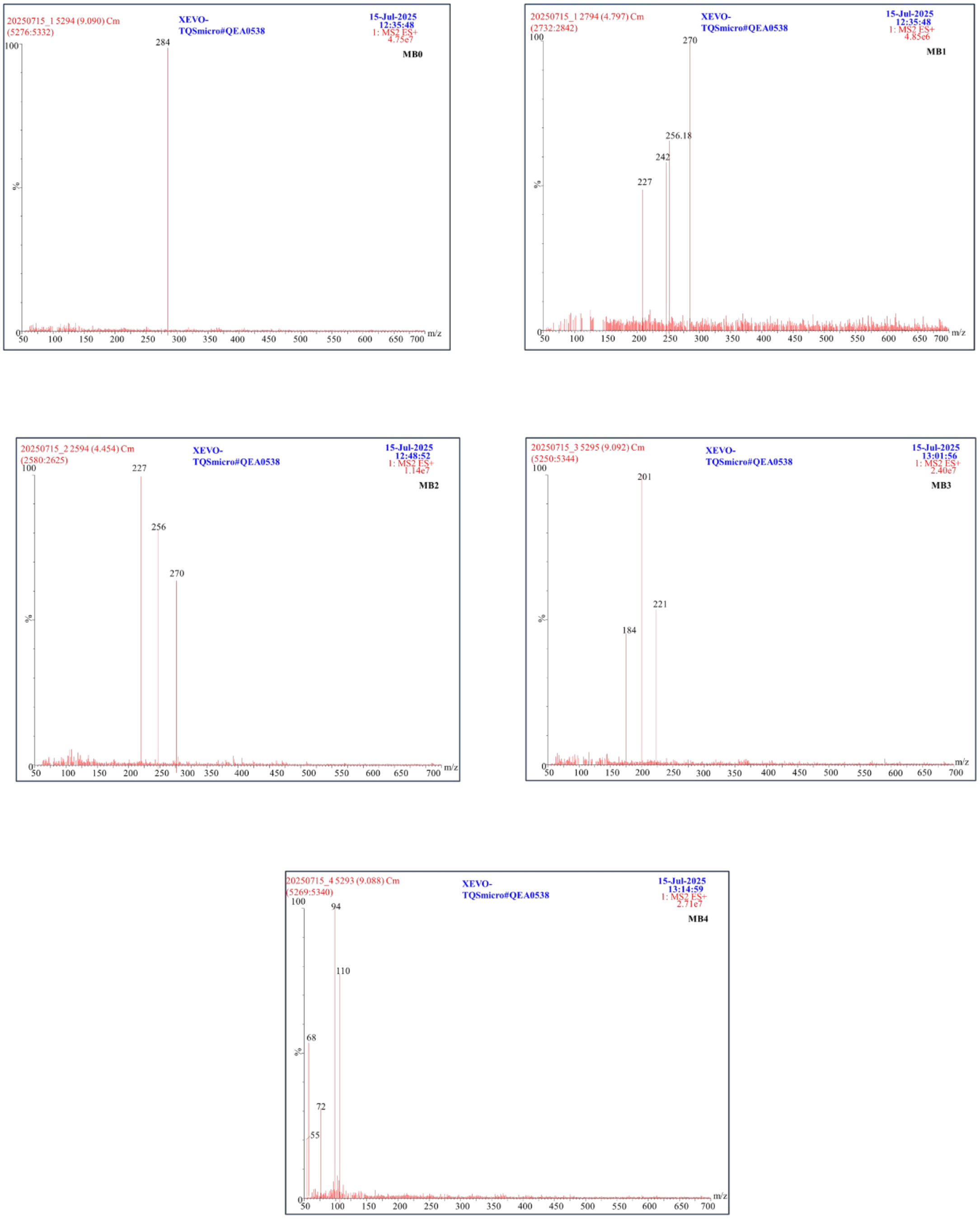

Figure S8. LC–MS spectra during MB photodegradation over the PPy–TiO_2–CNT photocatalyst: **(a)** MB0 (0 min), **(b)** MB1 (15 min), **(c)** MB2 (30 min), **(d)** MB3 (45 min), **(e)** MB4 (60 min).

References

(1) Li, S.; Chen, M.; He, L.; Xu, F.; Zhao, G. Preparation and Characterization of Polypyrrole/TiO2 Nanocomposite and Its Photocatalytic Activity under Visible Light Irradiation. *J. Mater. Res.* **2009**, *24* (8), 2547–2554.

(2) Sangareswari, M.; Meenakshi Sundaram, M. Development of Efficiency Improved Polymer-Modified TiO2 for the Photocatalytic Degradation of an Organic Dye from Wastewater Environment. *Appl. Water Sci.* **2017**, *7* (4), 1781–1790.

(3) Mousavi, S. M.; Rahmani, M. B. Enhanced Photocatalytic Performance of Semicrystalline Cl-Doped PPy@TiO2 Nanocomposites for MB Degradation. *Polym. Adv. Technol.* **2025**, *36* (8).

(4) Buenviaje, S. C. Jr.; Usman, K. A. S.; Edañol, Y. D. G.; Maylem, G. P.; Payawan, L. M. Jr. One-Pot Photochemical Synthesis of Solution-Stable TiO_2-Polypyrrole Nanocomposite for the Photodegradation of Methyl Orange. *Key Eng. Mater.* **2020**, *853*, 217–222.

(5) Silvestri, S.; Burgo, T. A. L.; Dias-Ferreira, C.; Labrincha, J. A.; Tobaldi, D. M. Polypyrrole-TiO2 Composite for Removal of 4-Chlorophenol and Diclofenac. *React. Funct. Polym.* **2020**, *146*, 104401.

(6) Pang, A. L.; Arsad, A.; Ahmadipour, M.; Azlan Hamzah, A.; Ahmad Zaini, M. A.; Mohsin, R. High Efficient Degradation of Organic Dyes by Polypyrrole-

Multiwall Carbon Nanotubes Nanocomposites. *Polym. Adv. Technol.* **2022**, *33* (5), 1402–1411.

(7) Buu, T. T.; Son, V. H.; Nam, N. T. H.; Hai, N. D.; Vuong, H.-T.; Quang, L. T. K.; Dat, N. M.; Lin, T. H.; Phong, M. T.; Hieu, N. H. Three-Dimensional ZnO–TiO2/Graphene Aerogel for Water Remediation: The Screening Studies of Adsorption and Photodegradation. *Ceram. Int.* **2023**, *49* (6), 9868–9882.

(8) Xue, J.; Zhang, J.; Yuan, M.; Lv, Y.; Chen, Z.; Wang, M. Visible-Light-Driven Au/PCN-224/Cu(II) Modified Fabric with Enhanced Photocatalytic Antibacterial and Degradation Activity and Mechanism Insight. *Sep. Purif. Technol.* **2024**, *333*, 125863.

(9) Houas, A. Photocatalytic Degradation Pathway of Methylene Blue in Water. *Appl. Catal. B* **2001**, *31* (2), 145–157.

(10) Kumar, A.; Ashraf, I. M.; Marnadu, R.; Shkir, M. Remarkable Enhancement of Photocatalytic Degradation of MB Dye from Aqueous Solution through Sr-Doped NiO NCs Nanosheets. *Ceram. Int.* **2025**, *51* (19), 29064–29078.
